# Keystone protist suppression triggers mesopredator release and biotic homogenization in complex soil microbial communities

**DOI:** 10.1093/ismejo/wraf253

**Published:** 2025-11-14

**Authors:** François Maillard, Fredrik Klinghammer, Briana H Beatty, Hanbang Zou, Enrique Lara, Edith C Hammer, Anders Tunlid, Peter G Kennedy

**Affiliations:** Department of Biology, Lund University, 223 62 Lund, Sweden; Department of Biology, Lund University, 223 62 Lund, Sweden; Department of Plant and Microbial Biology, University of Minnesota, St. Paul, MN 55108, United States; Department of Biology, Lund University, 223 62 Lund, Sweden; Department of Mycology, Real Jardín Botánico, Consejo Superior de Investigaciones Científicas, 28014 Madrid, Spain; Department of Biology, Lund University, 223 62 Lund, Sweden; Department of Biology, Lund University, 223 62 Lund, Sweden; Department of Plant and Microbial Biology, University of Minnesota, St. Paul, MN 55108, United States

**Keywords:** keystone species, mesopredator release, biotic homogenization, trophic cascade, bacteria, fungi, protists, brown food web, microfluidics

## Abstract

The keystone species concept holds that certain members of an ecological community, despite their low abundance, exert disproportionately large effects on species diversity and composition. In microbial ecology, experimental validation of this concept has been limited because targeted removal of individual species remains technically challenging. Here, we developed a procedure to test the keystone species concept within a soil microbial food web by selectively suppressing a protist predator at the microscale via phototoxicity in a microfluidic soil chip system. We targeted a hypotrich ciliate (subclass Hypotrichia), and combined microscopy with high-throughput amplicon sequencing of microbial taxonomic markers to assess, across multiple trophic levels, how its suppression affected microbial community abundance, diversity, and composition. Over the 20-day incubation, the chip system supported complex communities of bacteria, fungi, and protists. Following Hypotrichia suppression, two distinct ecological responses were observed: first, an increase in the relative abundance of flagellates, consistent with mesopredator release, accompanied by a significant rise in overall protist diversity; second, a convergence in protist community composition, indicative of biotic homogenization. Bacterial community abundance, richness, and composition remained unchanged, likely due to compensatory predation from a relative increase in bacterivorous flagellates. In contrast, fungal diversity decreased, presumably because the altered protist community favored facultative fungal consumers. Collectively, these findings provide direct experimental evidence that low-abundance microbial predators can function as keystone species, modulating predator community composition and diversity, and exerting cascading effects on lower trophic levels within microbial brown food webs.

## Introduction

More than 50 years ago, Robert Paine introduced the keystone species concept, showing that certain species, despite low relative abundance, can exert a disproportionate influence on community structure and stability [1, 2]. Since then, the idea has been explored widely for animals and plants, providing a framework for predicting community diversity and composition and for linking those patterns to biodiversity and ecosystem functioning [3–5]. In practice, following Paine [1] and Power *et al.* [3], the keystone species concept is demonstrated by showing that a low-abundance species can induce statistically significant shifts in community diversity, composition, or trophic structure. For example, in terrestrial and aquatic food webs, low-abundance apex predators often function as keystone species, exercising strong top–down control that propagates through trophic levels and frequently stabilizes entire communities [1–3, 6]. More recently, this concept has been extended from macroorganisms to microorganisms, with increasing application in microbial ecology [7–10].

In animal microbiome studies, certain bacterial taxa have been labeled as keystone species because their metabolic or co-metabolic activities underpin essential, community-wide biochemical processes despite their low abundance [9, 11–13]. The concept has likewise been applied to environmental microbiomes across aquatic and terrestrial systems, including soils. A literature survey by Banerjee *et al.* [10] compiled nearly 200 putative microbial keystone species, most of them bacterial. Yet, Röttjers and Faust [14] warned that the vast majority of microbial keystone candidates have been identified only by correlation-based approaches (i.e. network analyses based on high-throughput amplicon sequencing of microbial taxonomic markers that treat highly connected “hubs” as keystones) without direct empirical support. Indeed, just 3.5% of these proposed microbial keystone species have been validated experimentally [10, 14], compared with animal ecology, where empirical studies prevail relative to modeling approaches [6]. The gap is mainly methodological: manipulative experiments such as selective species removal, which is considered the gold standard for identifying keystones [3], are feasible with macroorganisms but remain technically challenging for microbial species embedded in complex communities.

Whereas animal ecology has often identified keystone species among predator rather than consumer trophic levels [3, 6], soil microbial studies have mostly highlighted decomposers as putative keystone species, mainly bacteria and, to a lesser extent, fungi [10, 15]. This emphasis is unexpected because belowground food webs (“microbial brown food webs”) [16] resemble aboveground macroscopic food webs in structure: they span multiple trophic levels, with microbial predators such as protists feeding on bacterial and fungal decomposers [17, 18]. Top–down control by these predators is therefore expected to help stabilize microbial communities, just as apex predators do at the macroscopic level [2, 3]. Across protist morphogroups (operational groupings by cell morphology and feeding mode—e.g. flagellates, amoebae, ciliates), ciliates are typically the largest but least abundant predators relative to flagellates and naked amoebae [19, 20]; their combination of large body size, low abundance, and high trophic position parallels the traits of apex predators known to play keystone roles in macroscopic systems, suggesting that certain ciliate species could serve a similar function in microbial brown food webs. Although network-based studies have proposed several soil protists as keystone candidates, these findings remain to be validated experimentally [21–23].

Here, we developed a new experimental procedure to test the keystone species concept in a microbial brown food web by selectively suppressing a protist predator embedded in a complex community containing other protist predators and bacterial and fungal decomposers. We used microfluidic soil chips that mimic natural soil microhabitats by creating synthetic pore spaces in continuous contact with the surrounding soil. These chips are rapidly colonized by diverse microbial communities, including bacteria, fungi, and protists, and function as newly formed pore networks that are continuously seeded by native soil biota [24, 25]. The platform also permits both real-time microscopy for direct counts of bacteria, fungi, and protists and the assessment of microbial community composition and diversity via environmental deoxyribonucleic acid (eDNA) extracted from the chip interior [24, 25]. Building on this system, we developed a phototoxicity-based microscale suppression technique in which a focused excitation beam at 400× magnification is applied to individual protist cells, inducing phototoxic death. We targeted the largest and most morphologically distinctive ciliate that consistently colonized chips inoculated with forest soil under our experimental conditions: a hypotrich ciliate (subclass Hypotrichia).

Hypotrichia have broad feeding ranges, but intermediate-sized species (≈ 80–100 μm) like our focal taxon feed mainly on bacteria and, to a lesser extent, on small protists such as flagellates; they do not prey on filamentous fungi, due to filter-feeding mechanisms that cannot capture or ingest fungal hyphae [26, 27]. We therefore expected the targeted Hypotrichia to be predominantly bacterivorous and hypothesized that its suppression would reshape decomposer communities through two trophic pathways. First, under a decomposer cascade scenario, relaxing Hypotrichia predation would increase bacterial abundance. The resulting intensification of bacterial competition would then suppress fungi, as they share overlapping ecological niches, thereby reducing fungal abundance and diversity [26, 28]. Second, following the mesopredator release framework of Ritchie and Johnson [29], removing the largest, low-abundance predator should free other protists that are its prey or competitors, allowing them to proliferate and thereby alter protist community composition while influencing diversity and evenness. We tested these predictions by pairing microscopy with eDNA-based approaches, including quantitative polymerase chain reaction (qPCR) and high-throughput amplicon sequencing of bacterial, fungal, and protist taxonomic markers, to track changes in microbial abundance, diversity, and community structure after Hypotrichia suppression.

## Materials and methods

### Experimental design

**Figure 1:**
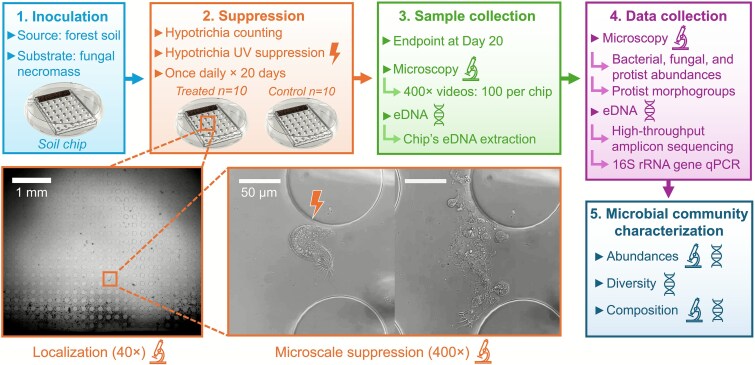
Experimental workflow used to test the effects of targeted Hypotrichia suppression on microbial communities: (i) soil chips were inoculated with fungal necromass as a carbon and nutrient source to mimic an organic matter rich patch, then forest soil was placed in direct contact with the chip entrance; (ii) for 20 days, Hypotrichia individuals were counted daily at 40× in both control and suppression chips (*n* = 10 per treatment) and subsequently eliminated at 400× in the suppression group; (iii) on day 20, 100 microscopy videos per chip were recorded and eDNA was extracted from each chip; (iv) microscopy data were used to quantify bacterial, fungal, and protist abundances and to assign protists to morphogroups, while eDNA enabled high-throughput amplicon sequencing of all three groups; (v) integrating microscopy and amplicon sequencing revealed how Hypotrichia suppression altered microbial abundance, composition, and diversity within the soil chips.

To test the keystone species concept, we established a microbial brown food web in microfluidic soil chips mirroring soil communities decomposing fungal organic matter. The chips remained connected to the surrounding soil to allow natural immigration, emigration, and recolonization. Within this setting, we applied daily, targeted, complete suppression of a putative keystone protist predator (Hypotrichia) for 20 days via phototoxicity, while leaving other taxa undisturbed. At the end of the incubation, we compared control and suppression chips using two approaches: microscopy-based counts of bacteria, fungi, and protists, and eDNA qPCR and amplicon sequencing of bacterial, fungal, and protist taxonomic markers (Fig. 1). We interpreted statistically significant treatment effects on community metrics (abundance, richness, diversity, composition) relative to the focal predator’s low abundance as evidence of a disproportionate keystone effect.

### Chip design and inoculation

Microfluidic chips had a cuboid design (14 720 μm × 5000 μm × 12 μm) with an array of ~100-μm-diameter circular pillars spaced 175 μm center-to-center (Fig. S1A), chosen for rapid inspection at 40× magnification and identification/suppression of specific protist taxa. Chips were fabricated following established protocols [25, 30]; full protocols are in the Supplementary Methods. Immediately after PDMS slab bonding—and taking advantage of the hydrophilicity induced by plasma treatment—100 μl of a 4% (mass/volume) autoclave-sterilized fungal necromass suspension was pipetted into the open chip entrance, allowing 10–30 μm necromass particles to enter by capillary action. Fungal necromass was selected as the primary carbon and nutrient source because it mimics organic matter encountered by microorganisms in forest topsoil, representing an ecologically relevant microsite where a mycelial network has recently senesced [31]. Both bacterial and fungal decomposers participate in necromass decomposition, promoting the development of diverse communities across decomposer domains [32]. We chose *Mortierella alpina* as the necromass source due to the ubiquity and abundance of this genus in soils [33]. *M. alpina* was cultured for 28 days in a 250-ml Erlenmeyer flask containing half-strength potato dextrose medium (pH 5). After 28 days, growth medium was replaced with distilled water, and the biomass was autoclaved. The autoclaved necromass was then washed with distilled water, freeze-dried, ground with a mortar and pestle, resuspended in sterile water, autoclaved again, and used to inoculate chips.

After necromass inoculation, chips were placed in contact with forest topsoil collected from a 60-year-old spruce (*Picea abies*) stand near Lund University’s Stensoffa field station (55.6928° N, 13.4540° E). The soil had pH 4.5 and was classified as sandy according to USDA texture classes [34]. Soil was sampled in November 2023 from the A horizon, pooled from five cores (0–10 cm), and sieved at 2 mm. Approximately 50 g of wet soil was subsampled and stored at 4°C until use. We selected this site because preliminary chip experiments with this soil consistently yielded a morphologically identifiable ciliate that could be unmistakably identified as a member of the subclass Hypotrichia (see below). In December 2023, 2 g of the pooled soil was placed directly at each chip entrance. The inoculated soil was moistened with sterile-filtered distilled water to saturation (Fig. S1B), Petri dishes were sealed with Parafilm and incubated in the dark at room temperature for 20 days. The soil in contact with the chip was remoistened after 10 days with filter-sterilized distilled water. This setup allowed the chip to function as a synthetic, transparent soil pore-space system interacting with the soil, facilitating natural microbial movement and colonization over the 20-day incubation. In total, 20 chips (labeled 1–20) were prepared and randomly assigned to a control group or a ciliate-suppression group (*n* = 10 each). Additionally, three chips were prepared on coverslips, placed in sealed Petri dishes without soil or necromass, and used as negative controls for potential eDNA contamination during sampling, DNA extraction, PCR, or sequencing.

### Hypotrichia suppression procedure

A protist morphotype, visually identified as belonging to the subclass Hypotrichia (Spirotrichea, Ciliophora), was selected because individuals of this morphotype constituted the largest protists colonizing the chips under our experimental conditions and, due to their low per-chip abundance compared with other protists (around 10 individuals per chip based on preliminary experiments within communities of thousands of protists), represented an ideal target to test the keystone species concept. The morphology of the observed individuals was distinct from other ciliate taxa and thus a readily identifiable target. Given the relatively moderate size of the observed Hypotrichia individuals (≈80–100 μm), we anticipated them to feed primarily on bacteria and to a lesser extent, potentially small flagellates [27] (Video S1). Under our experimental conditions, Hypotrichia were the largest protists observed, as the ~80 μm × 10 μm chip entrance restricted the entry of larger ciliates and most testate amoebae. Moreover, the chip entry size mimicked sandy soil pore sizes encountered in soil pore systems from the sandy soil selected for this experiment [35].

Over the 20-day incubation, both control and suppression chips (n = 10 per treatment) were inspected daily at 40× to count Hypotrichia individuals. All observations and imaging were performed on an inverted microscope (Nikon Ti2-E with PFS4 hardware autofocus, full 25-mm field of view, CoolLED pE300-White MB illumination connected via a 3-mm liquid light guide, and a Nikon Qi2 camera with a 1× F-mount adapter). For the suppression treatment, which was applied daily to all Hypotrichia present in the suppression chips, individuals were first located at 40×, then examined at 400× and exposed to excitation light (460 ± 20 nm; CoolLED pE300-White MB) at 100% intensity for 5 s. This exposure triggered cell death, defined here as membrane rupture in Hypotrichia individuals, likely via photoreactive compounds inducing oxidative stress and compromising membrane integrity. A test experiment on 97 protists of various sizes showed that this treatment affected only large cells (>40 μm) (see Supplementary Methods and Fig. S2). Combined with the small fraction of chip surface exposed per event (~0.05%), the procedure constituted a negligible disturbance to surrounding microbial communities. Hypotrichia were individually monitored by bright-field microscopy until cell breakdown was evident (Fig. S3; Video S2). If death did not occur after the initial exposure, an additional 2 s exposure was applied and the procedure repeated until suppression was observed. Photoexposed Hypotrichia formed vacuoles, indicating that cytoplasmic contents were not fully released into the chip, thereby minimizing potential bias due to providing additional resources to bacteria and fungi (Fig. S3). Our experiment thus mirrors the classic design of macroscopic keystone species experiments, which involve complete removal in open systems: discrete (daily) removal of all Hypotrichia cells coupled with natural recolonization from the connected soil matrix. Five chips were excluded from microscopy analyses due to technical issues or low Hypotrichia colonization; see Supplementary Methods and Table S1.

### Microscopy and image analysis of the chips

At 20 days post-inoculation, we recorded 15 s videos at 400× magnification using a digital camera (USB29 UXG M) to count bacteria, fungi, and protists. Videos captured movement, improving protist identification and distinguishing them from debris. For each chip, 100 videos (15 s each) were recorded from open spaces between pillars; specifically, 20 videos were taken per row at intervals of every five pillars from the entrance to ensure representative coverage. Videos were recorded and randomly coded by F.M., and counting of bacteria, fungi, and protists was performed by F.K. under a single-blind setup to reduce observational bias. To determine bacterial abundance, we used a 1–5 scoring system (1 = very low bacterial density, 5 = very high bacterial density; Fig. S4). Following Zou *et al.* [24] (see Supplementary Methods), we also applied a deep-learning segmentation model to automate bacterial counts. Bacterial density (cells per frame) was then summed across the 100 images per chip (Fig. S5).

Fungal hyphae were counted as single units when observed crossing the frame independently, to avoid overcounting branching hyphae. Protists were counted and categorized into morphogroups: ciliates (moving by cilia), amoebae (exhibiting amoeboid movement), and flagellates (moving via flagella; amoeboflagellates such as cercomonads were grouped with flagellates) (Fig. S6). Although this morphogroup classification does not necessarily reflect evolutionary relationships (except for ciliates), it provides a basis for inferring potential functional roles (e.g. flagellates as primarily bacterial feeders, amoebae as more omnivorous) and has been widely used in the literature, allowing direct comparisons with published research [19, 20].

### Chip environmental deoxyribonucleic acid extraction and microbial high-throughput amplicon sequencing and quantitative polymerase chain reaction

After video recording on day 20, residual soil was removed and the chip and its coverslip were cleaned to reduce eDNA contamination. The chip was then detached and processed for genomic DNA extraction using the PowerSoil Pro Kit (Qiagen, Hilden, Germany) following the manufacturer’s protocol (full details in the Supplementary Methods). Bacterial abundance was quantified by qPCR targeting the 16S ribosomal ribonucleic acid (rRNA) gene (primers 968F/1401R [36]) on a Stratagene Mx3005P system. Similar efforts were made with fungal qPCR (primers FR1/FF390 [37]), but were unsuccessful due to low fungal DNA yields. Microbial community structure was characterized by high-throughput sequencing of taxonomic markers from bacteria (16S V4 region using 515F–806R [38]), fungi (ITS2 region using 5.8S-Fun/ITS4-Fun [39]), and protists (18S rRNA gene using 616*f–1132r [40]) from 16 chip samples and three non-inoculated chip controls. Sequencing reads were processed using the DADA2 pipeline in R [41], including quality filtering, chimera removal, operational taxonomic unit (OTU) clustering at 97% similarity, and taxonomic assignment using the SILVA 138.1 [42], UNITE v10 (release 2024-04-04) [43], and PR2 v5.0.0 [44] databases, with additional filtering to remove contaminants and non-target sequences. To isolate indirect community responses from the direct effect of the manipulation, we removed all Hypotrichia OTUs from both control and suppression datasets before normalization. A summary of retained chips is provided in Table S1, and protist OTUs were regrouped into morphogroups based on taxonomy (Table S2). Full protocols are available in the Supplementary Methods.

### Data analysis

Analyses and visualizations were conducted in R [45] (α = 0.05). To assess the effect of the suppression procedure on Hypotrichia abundance over time, we fitted linear mixed-effects models (lme4 [46]) with “chip” as a random factor to account for repeated measures; fixed effects were evaluated using type III analysis of variance (ANOVA) with Satterthwaite’s method (lmerTest [47]). OTU richness, Simpson’s diversity, and Simpson’s evenness for bacterial, fungal, and protist communities were calculated using the vegan package [48]. For comparisons of microbial abundance and diversity metrics, normality was tested using the Shapiro–Wilk test; non-normal variables were log-transformed. Homoscedasticity was assessed using Bartlett’s test; if variances were homogeneous, a t-test was used; otherwise, a Welch’s t-test was applied. The effects of Hypotrichia suppression on community composition were analyzed by permutational ANOVA (PERMANOVA) (vegan) based on Bray–Curtis dissimilarities, with results visualized via non-metric multidimensional scaling (NMDS). Pearson’s correlation was used to assess relationships between bacterial, fungal, and protist diversity and Hypotrichia counts at day 20 post-inoculation, as well as between the abundance (microscopy) and relative abundance (amplicon sequencing) of flagellates, amoebae, and non-Hypotrichia ciliates and Hypotrichia counts.

## Results

**Figure 2:**
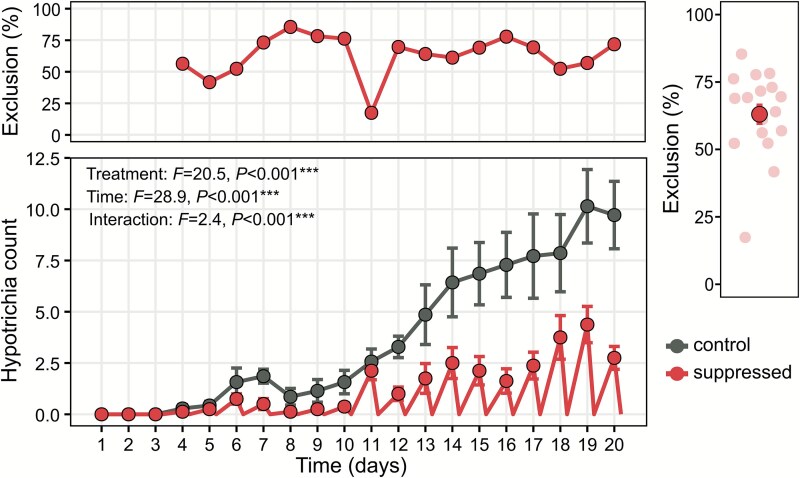
Effects of the suppression procedure on Hypotrichia abundance. Daily Hypotrichia individual counts per chip over 20 days (averaged by treatment: control vs. suppressed). In the suppressed treatment (red line), all the counted Hypotrichia were suppressed. The effects of treatment, time (days post-inoculation), and their interaction were evaluated using a linear mixed-effects model (time as categorical, chip as random factor) via type III ANOVA with Satterthwaite’s method (^*^P < .05, ^**^P < .01, ^***^P < .001). The top panel shows average Hypotrichia suppression percentage over time, and the right panel shows average suppression percentage across all time points. Data are mean ± SE.

Microscopy at 40× magnification revealed that Hypotrichia individuals began colonizing the chips by day 4 post-inoculation (Fig. 2). In control chips, their numbers increased significantly over time (*F* = 28.9, *P* < .001), reaching peak abundance by day 19 (Fig. 2). The suppression treatment significantly reduced Hypotrichia abundance (*F* = 20.5, *P* < .001), with exclusion efficiencies ranging from 23% to 75% and averaging 63% across the experiment. Amplicon sequencing of protist communities at day 20 showed that Hypotrichia relative abundance declined from 21.1% in controls to 12.7% in the suppression treatment (~40% reduction), although this difference was not statistically significant (Fig. S7A). Six distinct Hypotrichia OTUs were identified, with three pairs exhibiting high sequence similarity (93%–95%; Fig. S7B). Searches against the PR2 database matched these OTUs to either Oxytrichidae or Pseudourostylidae. Specifically, OTU30 was dominant, accounting for 74% of Hypotrichia sequences and appearing in 69% of chips, whereas most other Hypotrichia OTUs were detected in only a single chip (Fig. S7C).

**Figure 3:**
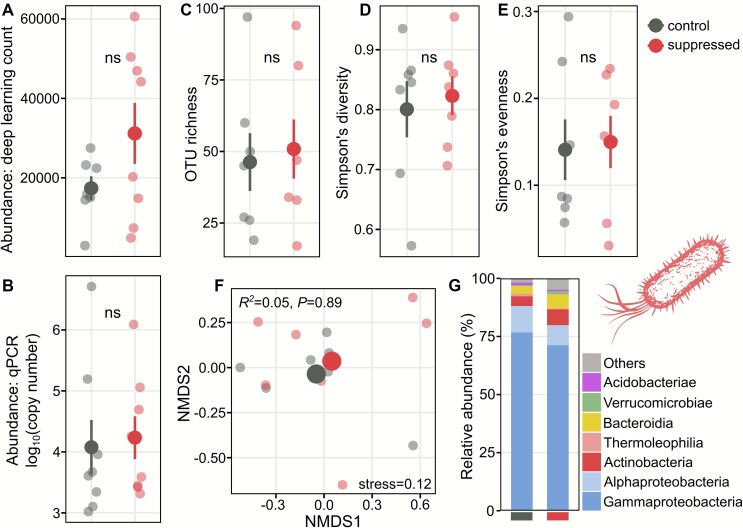
Effects of Hypotrichia suppression on bacterial communities. (A) Bacterial abundance based on microscopy combined with a deep-learning bacterial cell identification and counting algorithm (summed from 100 images per chip), (B) bacterial abundance based on 16S rRNA gene qPCR per chip, (C) OTU richness, (D) Simpson’s diversity, and (E) Simpson’s evenness from 16S rRNA gene amplicon sequencing. Large dark dots indicate means ± SE; lighter dots represent individual chips. Statistical comparisons were made using *t*-tests or Welch’s tests as appropriate. (F) Bacterial community composition (rarefied OTU abundance) shown by NMDS using Bray–Curtis dissimilarity. Large dots are centroids; small dots individual communities. PERMANOVA tested treatment effects (^*^P < .05, ^**^P < .01, ^***^P < .001). (G) Relative abundance of the seven most abundant bacterial classes averaged by treatment.

Bacterial abundance, assessed via deep-learning analysis of microscopy videos (400× magnification; 100 videos per chip) on day 20 post-inoculation, increased by an average of 80% in Hypotrichia-suppressed chips, although this difference was not statistically significant (Fig. 3A), matching human-based visual scoring (Fig. S8) and bacterial qPCR results (Fig. 3B). Bacterial OTU richness (Fig. 3C), Simpson’s diversity (Fig. 3D), and evenness (Fig. 3E) were also not significantly affected by Hypotrichia suppression. The NMDS analysis of bacterial community composition further revealed no clear treatment-related clustering, a result supported by the non-significant PERMANOVA (Fig. 3F). Gammaproteobacteria dominated the bacterial communities (up to 70% of sequences), followed by Alphaproteobacteria, Actinobacteria, and Bacteroidia, with no marked class-level differences between treatments (Fig. 3G). The dominant bacterial genera included *Cavicella*, *Alkanindiges* (both Gammaproteobacteria), *Burkholderia* (Betaproteobacteria), *Mycobacterium* (Actinobacteria), *Roseateles* (Betaproteobacteria), *Chitinophaga*, *Mucilaginibacter* (both Bacteroidota), and *Herminiimonas* (Betaproteobacteria) (Fig. S9).

**Figure 4:**
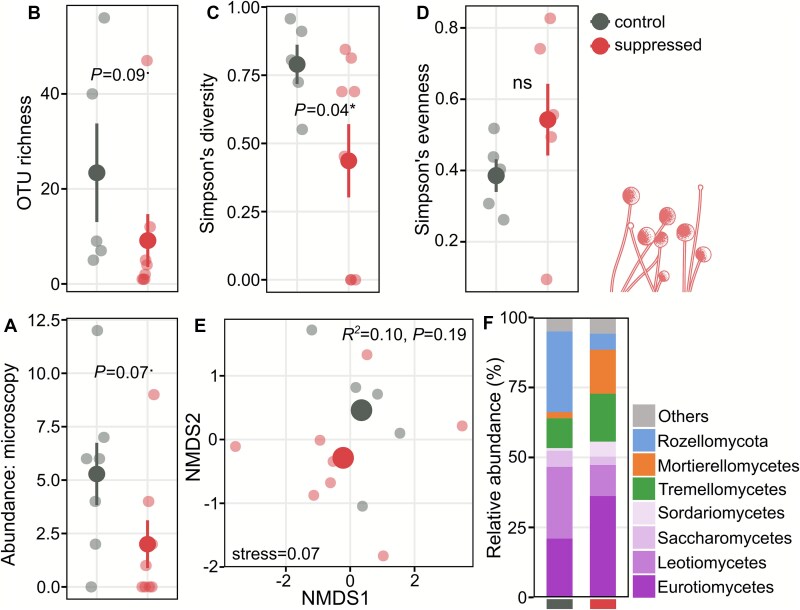
Effects of Hypotrichia suppression on fungal communities. (A) Fungal abundance from microscopy (summed from 100 images per chip), (B) OTU richness, (C) Simpson’s diversity, and (D) Simpson’s evenness based on ITS amplicon sequencing. Large dark dots indicate means ± SE; lighter dots represent individual chips. Statistical comparisons were made using *t*-tests or Welch’s tests as appropriate. (E) NMDS analysis (Bray–Curtis dissimilarity) of fungal OTU composition (large dots are centroids; small dots individual chips). Treatment effects were compared by PERMANOVA (^*^P < .05, ^**^P < .01, ^***^P < .001). (F) Relative abundance of the seven most abundant fungal classes averaged by treatment.

Fungal abundance, assessed on day 20 by microscopy (400× magnification, summed from 100 videos per chip), was lower in the Hypotrichia-suppression treatment than in controls, although this difference was only marginally significant (*P* = .07; Fig. 4A). Amplicon sequencing analyses likewise indicated a marginally significant reduction in fungal OTU richness (*P* = .07) and significantly lower Simpson’s diversity in the suppressed treatment (*P* = .04; Fig. 4B and C), with no significant difference in evenness (Fig. 4D). NMDS analysis of fungal OTU composition showed some separation of treatment centroids, although the PERMANOVA did not detect a significant difference (Fig. 4E). The fungal community included Ascomycota (Eurotiomycetes and Leotiomycetes), Basidiomycota (Tremellomycetes yeasts), and early-diverging lineages (Mortierellomycetes and Rozellomycota; Fig. 4F). Dominant genera spanned filamentous and yeast forms, including *Aspergillus* and *Penicillium* (Eurotiomycetes), *Oidiodendron* (Leotiomycetes), *Solicoccozyma* and *Saitozyma* (Tremellomycetes), *Mortierella* (Mortierellomycota), and *Nadsonia* (Saccharomycetes; Fig. S10).

**Figure 5:**
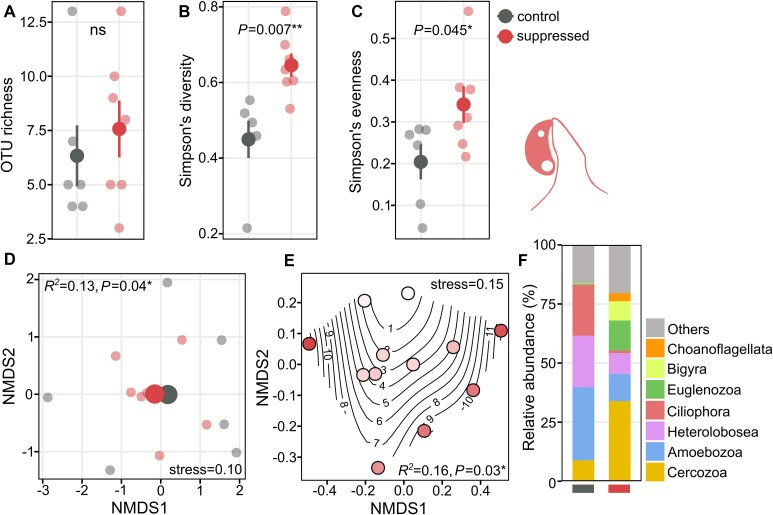
Effects of Hypotrichia suppression on protist communities based on 18S rRNA gene amplicon sequencing. (A) OTU richness, (B) Simpson’s diversity, and (C) Simpson’s evenness (large dots means ± SE; small dots individual chips; statistical comparisons by *t*-tests or Welch’s tests). (D) Protist community composition (NMDS with Bray–Curtis dissimilarity; large dots centroids, small dots individual communities). (E) NMDS with Hypotrichia counts at day 20 as a continuous variable (circles colored by Hypotrichia abundance, darker indicates higher abundance; segments indicate specific counts per chip). Treatment effects tested via PERMANOVA (^*^P < .05, ^**^P < .01, ^***^P < .001). (F) Relative abundance of the seven most abundant protist phyla/classes averaged per treatment.

Protist OTU richness at day 20 (amplicon sequencing) did not differ significantly between treatments (Fig. 5A). However, both Simpson’s diversity (*P* = .007; Fig. 5B) and evenness (*P* = .045; Fig. 5C) were significantly higher in the suppression treatment. PERMANOVA also indicated a significant effect of Hypotrichia suppression on protist OTU composition (*P* = .04). Consistent with this, the NMDS showed greater among-chip variability in control communities, whereas suppressed chips exhibited more homogeneous communities (Fig. 5D). Treating Hypotrichia abundance as a continuous variable (40× microscopy counts at day 20) confirmed the pattern: lower Hypotrichia counts were associated with greater similarity in protist community composition among chips (Fig. 5E). As noted in the methods, these analyses were based on datasets from which we removed all Hypotrichia OTUs from both control and suppression treatments before normalization to isolate indirect protist community responses from the direct effect of the Hypotrichia suppression procedure. Analyses including Hypotrichia OTUs yielded similar results, with significant differences in Simpson’s diversity (*P* = .023), evenness (*P* = .022), and OTU composition (*P* = .040) (Fig. S11). The control chips were dominated by Amoebozoa, Heterolobosea, Ciliophora, and Cercozoa, while suppressed chips had substantial abundances of Euglenozoa, Bigyra, and Choanoflagellata (Fig. 5F). Other microbial eukaryotes, excluded from the above analyses, were also detected by 18S rRNA gene amplicon sequencing, specifically, Rotifera occurred at low relative abundance and only in a single sample (Fig. S12), whereas nematode sequences represented ~40% of the total 18S rRNA gene sequences, with no significant differences between treatments (Fig. S12).

**Figure 6:**
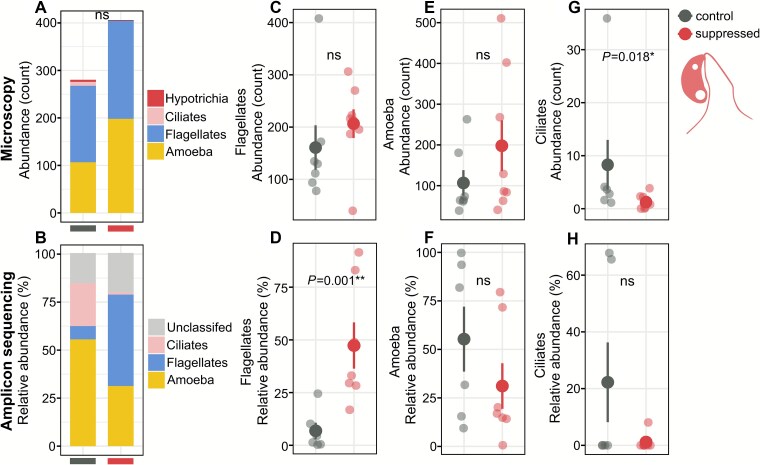
Effects of Hypotrichia suppression on protist abundance based on microscopic analysis. (A) Total protist count (summed from 100 images per chip; Hypotrichia not included in “ciliates”), (C) flagellate abundance, (E) amoeba abundance, and (G) ciliate abundance. Effects of Hypotrichia suppression on (B) relative abundance of OTUs and specifically for (D) flagellates, (F) amoebae, and (H) ciliates based on 18S rRNA gene amplicon sequencing. Statistical comparisons performed via *t*-tests or Welch’s tests (^*^P < .05, ^**^P < .01, ^***^P < .001).

Microscopy-based counts (400× magnification, summed from 100 videos per chip) indicated an average protist abundance of ~280 individuals in controls versus ~400 in suppressed treatments per 100 microscopy frames, though this difference was not statistically significant (Fig. 6A). Amoebae and flagellates dominated both the microscopy (Fig. 6A) and amplicon sequencing datasets (Fig. 6B). Flagellate relative abundance increased significantly with Hypotrichia suppression (microscopy: Fig. 6C; amplicon sequencing: *P* = .001, Fig. 6D), while amoebae abundance was not significantly affected in either analysis (microscopy: Fig. 6e; amplicon sequencing: Fig. 6F). Conversely, non-Hypotrichia ciliates decreased significantly in the microscopy analyses (*P* = .018), though the trend was non-significant in the amplicon sequencing data (Fig. 6G and H). At finer taxonomic resolution, dominant flagellate taxa (e.g. *MAST-12C*, *Neobodo*, *Monosiga*, *Cercomonas*, *Bodo*, and *Glissomonadida*) increased with suppression, whereas dominant non-Hypotrichia ciliates (*Platyophrya*, *Microthoracida*) decreased. Amoebae showed variable, taxon-specific responses (Fig. S13).

**Figure 7:**
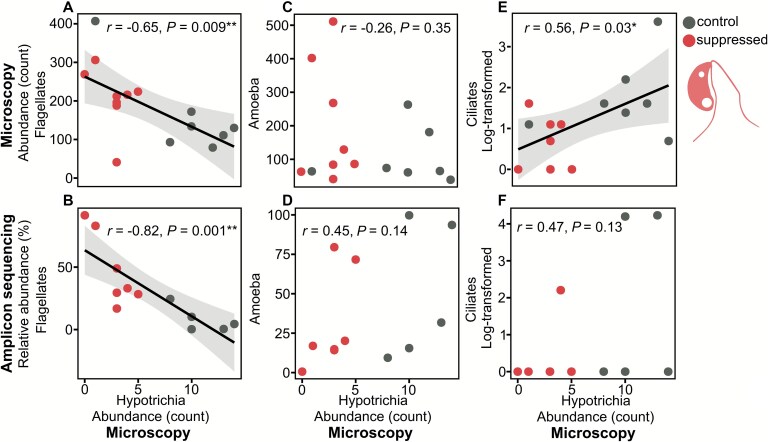
Pearson’s correlations between protist groups and Hypotrichia abundance (day 20): (A) flagellate abundance (microscopy), (B) flagellate relative abundance (18S rRNA gene amplicon sequencing), (C) amoeba abundance (microscopy), (D) amoeba relative abundance (18S rRNA gene amplicon sequencing), (E) ciliate abundance (microscopy), (F) ciliate relative abundance (18S rRNA gene amplicon sequencing). Significance marked by asterisks (^*^P < .05, ^**^P < .01, ^***^P < .001).

When analyzed as a continuous variable, Hypotrichia abundance at day 20 showed no significant relationship with bacterial (Fig. S14A) or fungal Simpson’s diversity (Fig. S14B), but was significantly negatively correlated with protist Simpson’s diversity (*P* = .03; Fig. S14C). Hypotrichia abundance was also significantly negatively correlated with flagellate abundance in both the microscopy (*P* < .01; Fig. 7A) and amplicon sequencing (*P* < .01; Fig. 7B) analyses. Amoebae showed no significant correlation in either analysis (microscopy: Fig. 7C; amplicon sequencing: Fig. 7D). Finally, non-Hypotrichia ciliates were significantly positively correlated with Hypotrichia abundance in the microscopy analysis (*P* = .03; Fig. 7E), but not in the amplicon sequencing analysis (Fig. 7F).

## Discussion

Complex interacting microbial communities were successfully established in the soil chips (i.e. synthetic soil pore-space systems), with average species richness of 48 bacterial OTUs, 14 fungal OTUs, and 7 protist OTUs. These communities spanned broad phylogenetic diversity—including Proteobacteria, Actinobacteria, and Bacteroidota among bacteria; Eurotiomycetes, Leotiomycetes, Tremellomycetes, and Mortierellomycetes among fungi; and Cercozoa, Amoebozoa, Heterolobosea, and Ciliophora among protists; all abundant lineages within soil microbial communities [32, 49–51]. Moreover, the relative abundances of protist morphogroups under our experimental conditions (flagellates > amoebae > ciliates) matched in situ observations from soil protist communities [19, 20]. Collectively, these results indicate that the microbial assemblages that developed in the chips accurately reflected the taxonomic and functional diversity and trophic structure of microbial communities colonizing organic-matter patches in soil.

Contrary to our first hypothesis that Hypotrichia suppression would increase bacterial abundance by reducing top–down control, we found that bacterial communities remained remarkably stable across treatments. We suspect this stability resulted from the compensatory increase in flagellate relative abundance observed following Hypotrichia suppression. Flagellates, which are known bacterial predators [52–54], may have occupied the predatory niche vacated by Hypotrichia, thereby maintaining top–down control on bacterial populations. Alternatively, bacterial communities under our experimental conditions may have been able to resist ciliate predation; consistent with that possibility, we observed bacterial biofilm formation in the chips, which may limit grazing [55]. Other studies have reported limited top–down control of bacteria by ciliates or flagellates [20, 56, 57], and Clarholm [58] specifically highlighted amoebae as more effective bacterial predators in soils. In support of our first hypothesis, albeit through a different mechanism, Hypotrichia suppression significantly decreased fungal diversity. Initially, we had expected fungal community changes to result from increased bacterial competition. However, bacterial abundance, richness, and composition were unchanged, suggesting other mechanisms drove the fungal diversity decline. We infer that the decline we observed in fungal diversity was most likely due to increased flagellate abundance linked with Hypotrichia suppression. Certain dominant flagellates that increased in response to Hypotrichia suppression (e.g. *Cercomonas*) have been described as facultative fungal predators [17, 59], suggesting that Hypotrichia suppression may have indirectly intensified flagellate top–down control on fungi, cascading into a decrease in fungal diversity. This cascading effect on a lower trophic level due to experimental suppression of a low-abundance predator, even one not serving as a direct consumer of the affected prey group, aligns well with the keystone species concept.

In clear support of our second hypothesis, we observed significant shifts in protist community composition and diversity following Hypotrichia suppression, prominently marked by increased flagellate relative abundance and the proliferation of lineages such as Bigyra, Choanoflagellata, and Euglenozoa. These findings parallel the mesopredator release concept in animal ecology, where removing apex predators leads to subordinate predator proliferation [29, 60]. The significant negative correlation between flagellates and Hypotrichia abundance, in both microscopy and community sequencing data, suggests competition for bacterial prey; however, direct Hypotrichia predation on flagellates cannot be excluded, as some Hypotrichia consume small flagellates [27, 61]. Despite their lower abundance relative to flagellates in our system, Hypotrichia, because of their large size and rapid movement, can traverse larger areas within the synthetic soil pore-space and encounter more bacterial cells than flagellates, potentially capturing more bacteria per unit time, aligning with reports of negative interactions, either through competition or predation, between ciliates and flagellates [62–64]. Hypotrichia suppression also resulted in more similar protist communities, a microscale example of biotic homogenization [65, 66] that is consistent with Paine’s original keystone studies [1, 2]. Although homogenization here coincided with increased protist diversity, these processes are not mutually exclusive and raise questions about broader cascading effects on community stability and function over time. Taken together, these observations support the keystone species concept in our system: a low-abundance predator strongly affecting community structure, whether the effects are beneficial or detrimental [1, 2].

Our 18S rRNA gene amplicon sequencing data indicated the likely presence of three distinct Hypotrichia species within the morphospecies targeted via microscopy, with one being overwhelmingly more abundant and prevalent than the others. The higher abundance of OTU30 relative to the other Hypotrichia OTUs indicates that our suppression largely targeted a single low abundance species. We also recognize that our Hypotrichia suppression was not a continuous, complete removal treatment, because of rapid recolonization from the surrounding soil. Sealing the chip entrance after inoculation, once Hypotrichia had colonized (around day 8 under our conditions), together with selective suppression, might have achieved complete suppression; however, this approach would reduce ecological relevance, as microbial communities undergo rapid succession during organic matter decomposition, and our chip design mimicked recently senesced fungal necromass [67]. Moreover, keeping the chips open preserved the natural connectivity of pore networks, maintaining realistic immigration–emigration dynamics, resource exchange, and predator–prey encounter rates characteristic of soil. Furthermore, our design mirrored classic keystone removal studies in open systems, in which recolonization is part of the process being tested and avoids isolation artifacts [1–3].

Although our study provides robust evidence that suppressing a low abundance microbial predator can induce major changes in microbial community composition and diversity, some limitations must be acknowledged and could be improved in future experiments. First, we observed high within-treatment variability despite using the same homogenized soil pool as inoculum. We suspect this variability reflects priority effects, whereby the identity of the first chip colonizers (bacteria, fungi, or protists) influenced subsequent community assembly [68]. This variability reduced statistical power, especially when a few chips showed unusual patterns—such as extremely low total protist counts or an early peak followed by a rapid decline in Hypotrichia in the control treatment. Establishing predefined exclusion criteria for outlier chips could help mitigate these issues in future experiments. A second limitation was the presence of nematodes, which, although not significantly different between treatments and primarily inactive or dead within the chips, dominated the 18S rRNA gene amplicon sequencing dataset and reduced protist read depth. Thus, designing a chip entrance that excludes nematodes will improve protist sequencing quality. The endpoint design for our molecular analyses (i.e. analysis at day 20) also did not capture temporal dynamics, which is a component of the keystone species concept [1–3], so incorporating time-series sampling will be important. Further, the rupture of Hypotrichia cells, even partially due to vacuole formation, may have released cellular contents and associated nutrients that could have altered trophic dynamics. However, given the limited number of Hypotrichia suppressed in the treated group linked with their low relative abundance compared with other protists and the provision of high-quality organic matter (fungal necromass) as the primary carbon and nutrient source, we suspect that any such effect was likely minor. Additionally, while phototoxicity effectively suppressed large protists such as ciliates, future developments pairing AI-driven recognition of protist morphologies with automated high-magnification suppression (e.g. 1000×) could enable efficient, species-specific, and temporally continuous suppression within synthetic soil pore-space systems. Finally, given that our protocol can suppress large amoebae, targeting amoebae in future keystone-species experiments will clarify the relative influence (“weight”) of protist predators within microbial brown food webs.

In terms of ecological relevance of the chip system, while matching sandy soil in pore size, our chips were fully water-filled during the experiment with all pores connected and lacked the presence of plant roots and associated mycorrhizal fungi in the connected soil matrix. As such, they may not fully reflect in situ soil functioning. Future work using more realistic chip architectures including potential refugia similar to the classic ecological studies of Huffaker [69] will be important to more fully understand how predator–prey relationships are structured in microbial brown food webs. Additionally, because we used fungal necromass as the carbon and nutrient source to create a brown food web, it will be informative to test other organic matter sources as well as no added organic matter to create more oligotrophic conditions, since Hypotrichia effects on microbial communities may be context-dependent. More broadly, integrating co-occurrence network analyses of in situ soil communities with the microscale suppression of targeted protist taxa used here represents a promising framework for identifying and experimentally validating keystone microbial predators.

## Conclusions

Here, we deployed a microfluidic soil chip system together with selective suppression of a protist predator by targeted microscale phototoxicity to experimentally test the keystone species concept within microbial communities. We combined microscopy and high-throughput amplicon sequencing of microbial taxonomic markers, which yielded broadly concordant results, opening opportunities to study soil microbial communities at both high taxonomic (amplicon sequencing) and functional (microscopy) resolution. Our findings validate key tenets of the keystone species concept, showing that a low abundance predator can disproportionately influence community diversity and composition. These results have two immediate implications: technically, they establish a platform for selectively suppressing protist predators, enabling the experimental identification of keystone species within microbial food webs; ecologically, they demonstrate that microbial predators, not just decomposers, can act as keystone species in soil communities.

## Supplementary Material

Figure_S1_wraf253

Figure_S2_wraf253

Figure_S3_wraf253

Figure_S4_wraf253

Figure_S5_wraf253

Figure_S6_wraf253

Figure_S7_wraf253

Figure_S8_wraf253

Figure_S9_wraf253

Figure_S10_wraf253

Figure_S11_wraf253

Figure_S12_wraf253

Figure_S13_wraf253

Figure_S14_wraf253

Table_S1_wraf253

Table_S2_wraf253

Video_S1_wraf253

Video_S2_wraf253

Dataset_1_wraf253

Dataset_2_wraf253

Dataset_3_wraf253

Dataset_4_wraf253

Supplementary_material_Legends_wraf253

Supplementary_Methods_wraf253

## Data Availability

Raw sequencing files (16S, ITS, and 18S) have been deposited in the NCBI Sequence Read Archive (SRA) under BioProject PRJNA1302515. All other data generated during this study are included in the supplementary information files.
