# Correction to: Keystone protist suppression triggers mesopredator release and biotic homogenization in complex soil microbial communities

**DOI:** 10.1093/ismejo/wrag161

**Published:** 2026-07-03

This is a correction to: François Maillard, Fredrik Klinghammer, Briana H Beatty, Hanbang Zou, Enrique Lara, Edith C Hammer, Anders Tunlid, Peter G Kennedy, Keystone protist suppression triggers mesopredator release and biotic homogenization in complex soil microbial communities, *The ISME Journal*, Volume 19, Issue 1, January 2025, wraf253, https://doi.org/10.1093/ismejo/wraf253

In the originally published version of this manuscript, the protocol erroneously stated that UV excitation at 395 nm was used for the phototoxic suppression of protists. Protist suppression was instead performed using blue-light excitation at 460 ± 20 nm.

In the abstract, the reference to ``[...] ultraviolet-induced phototoxicity in a microfluidic soil chip system'' has been corrected to read ``[...] phototoxicity in a microfluidic soil chip system''.

In the `Introduction' section, a sentence has been corrected to read: ``Building on this system, we developed a phototoxicity-based microscale suppression technique in which a focused excitation beam at 400× magnification is applied to individual protist cells, inducing phototoxic death''

Instead of: ``Building on this system, we developed an ultraviolet (UV)-based microscale suppression technique in which a focused UV-excitation beam at 400× magnification is applied to individual protist cells, inducing phototoxic death''.

In the `Materials and methods' section, the exposure has been corrected to read: ``excitation light (460 ± 20 nm; CoolLED Pe300-White MB) at 100% intensity for 5s'' instead of: ``UV-range excitation light (395 nm; CoolLED pE300-White MB) at 100% intensity for 5s''.

These corrections concern only the phototoxic suppression procedure and do not affect the results, interpretation, or conclusions of the Article.

Additionally, the Supplementary Methods file and the Supplementary Figure Legends file were inadvertently omitted from the originally published version. These have been added as part of the supplementary material.

