# Supplementary figures and images for: Keystone protist suppression triggers mesopredator release and biotic homogenization in complex soil microbial communities
Source: ISME J. 2025 Nov 14;19(1):wraf253. doi: 10.1093/ismejo/wraf253 (PMC12676721; doi:10.1093/ismejo/wraf253)

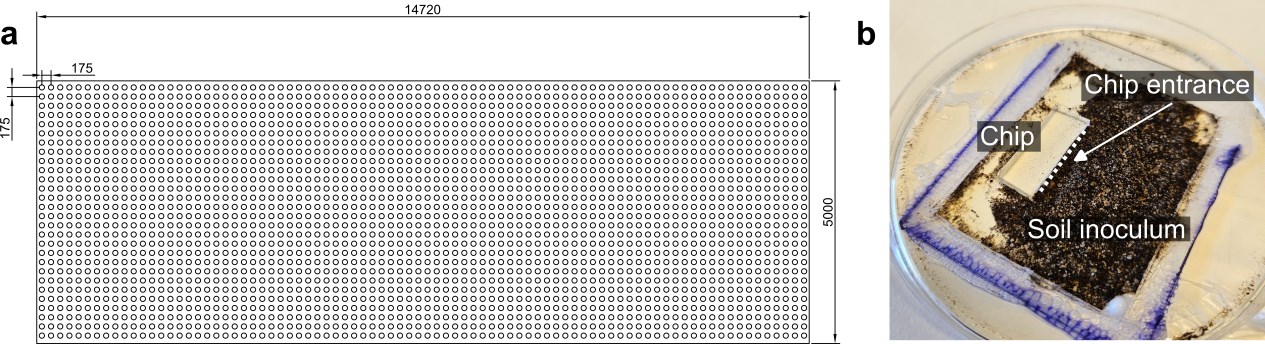

Supplement: Figure_S1_wraf253 [file figure_s1_wraf253.jpeg]

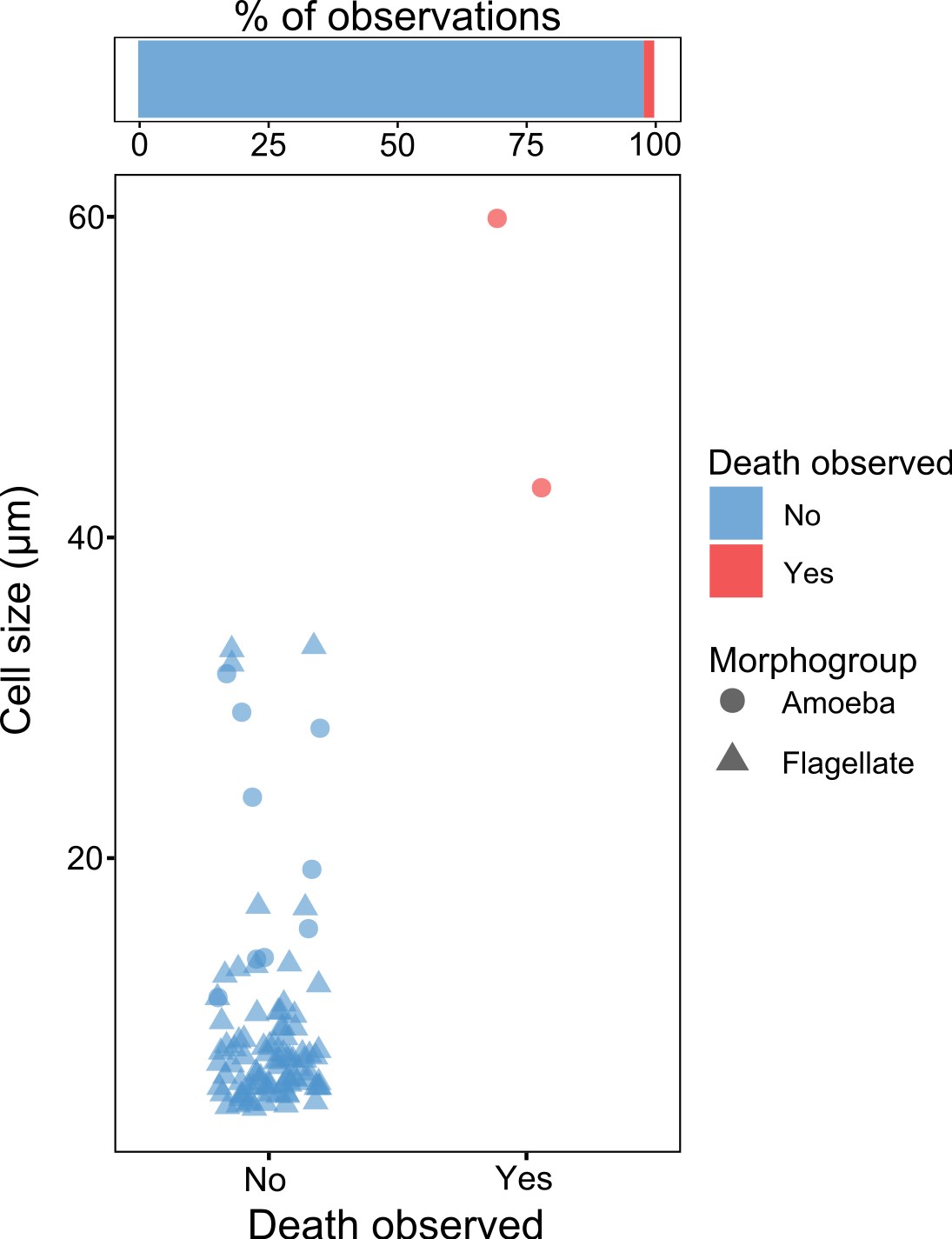

Supplement: Figure_S2_wraf253 [file figure_s2_wraf253.jpeg]

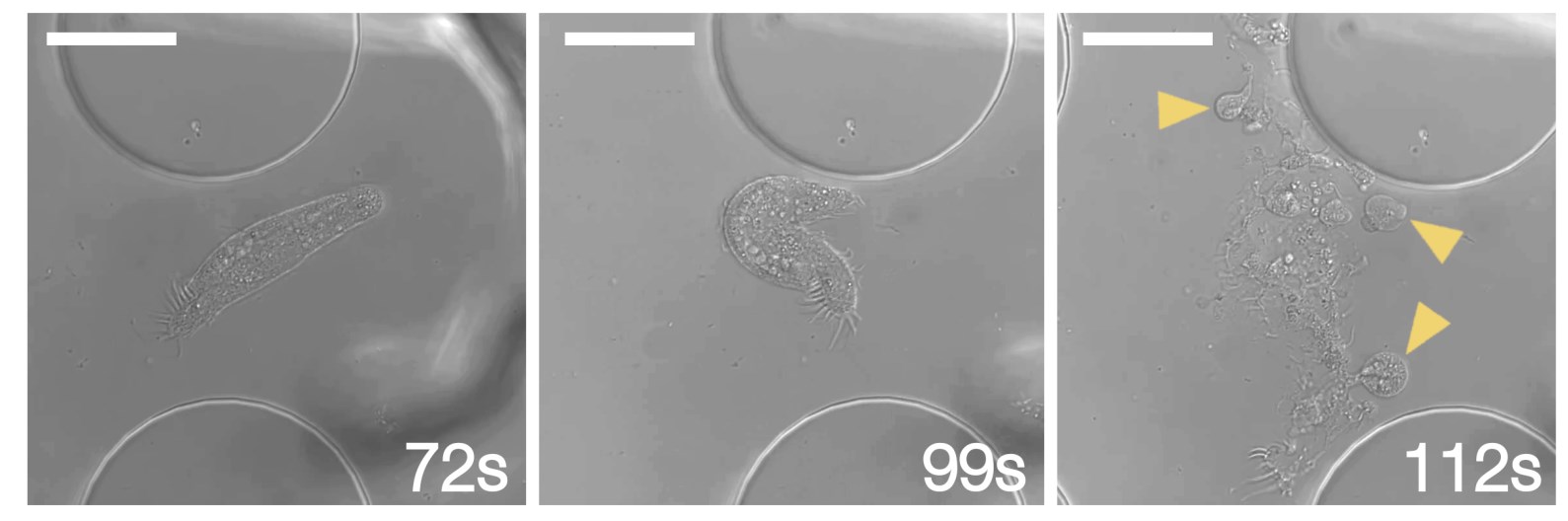

Supplement: Figure_S3_wraf253 [file figure_s3_wraf253.jpeg]

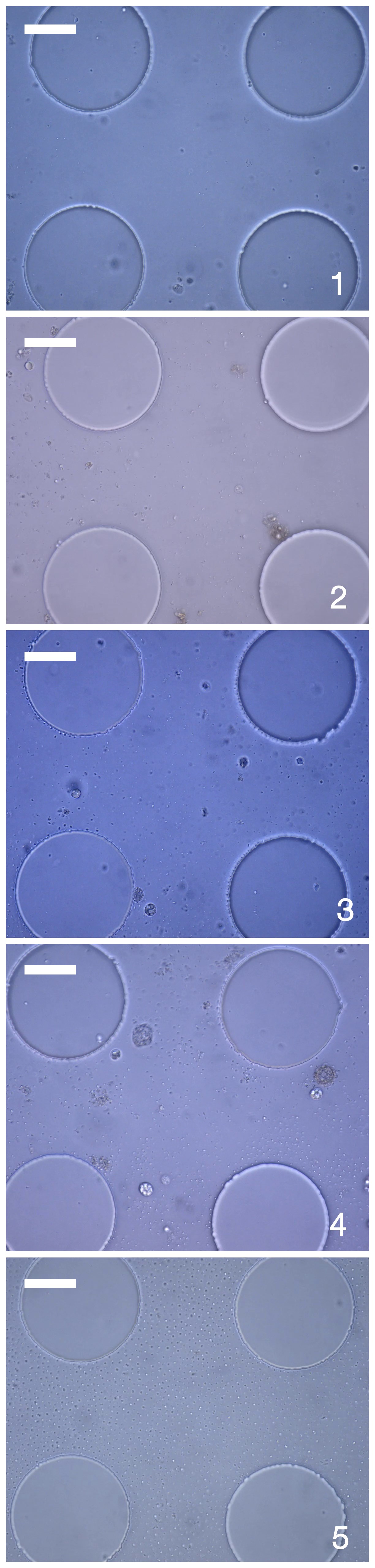

Supplement: Figure_S4_wraf253 [file figure_s4_wraf253.jpeg]

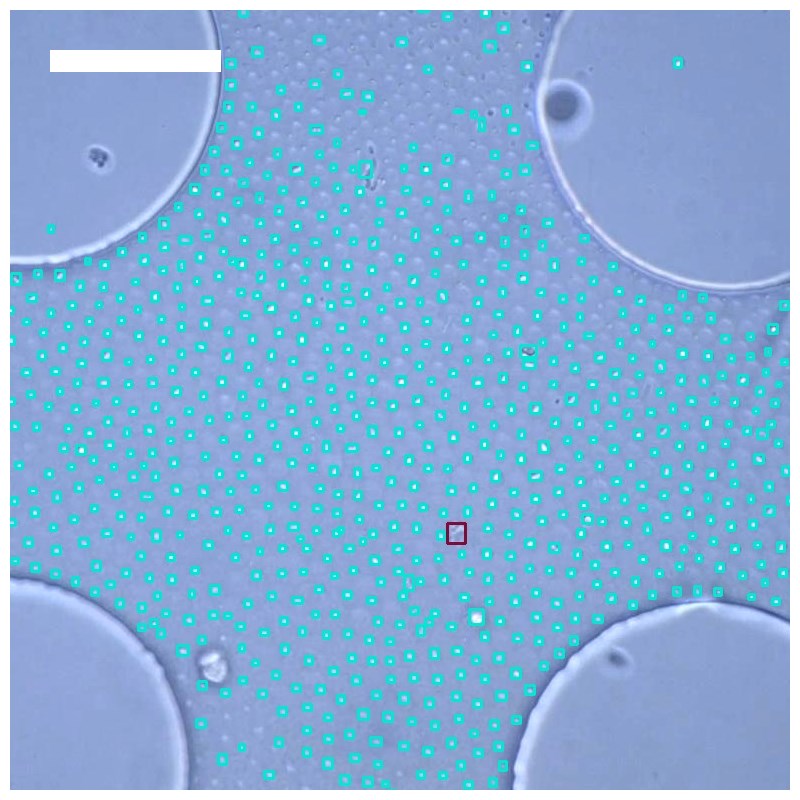

Supplement: Figure_S5_wraf253 [file figure_s5_wraf253.jpeg]

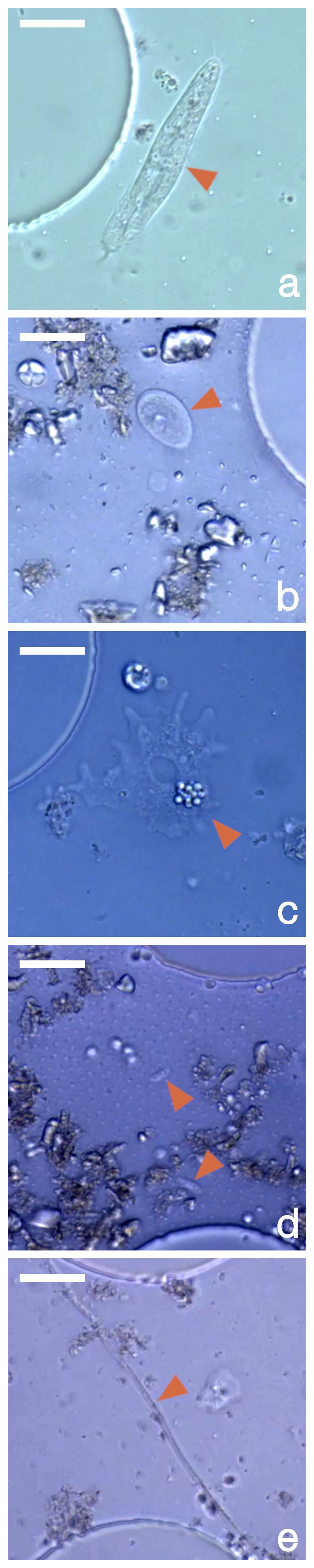

Supplement: Figure_S6_wraf253 [file figure_s6_wraf253.jpeg]

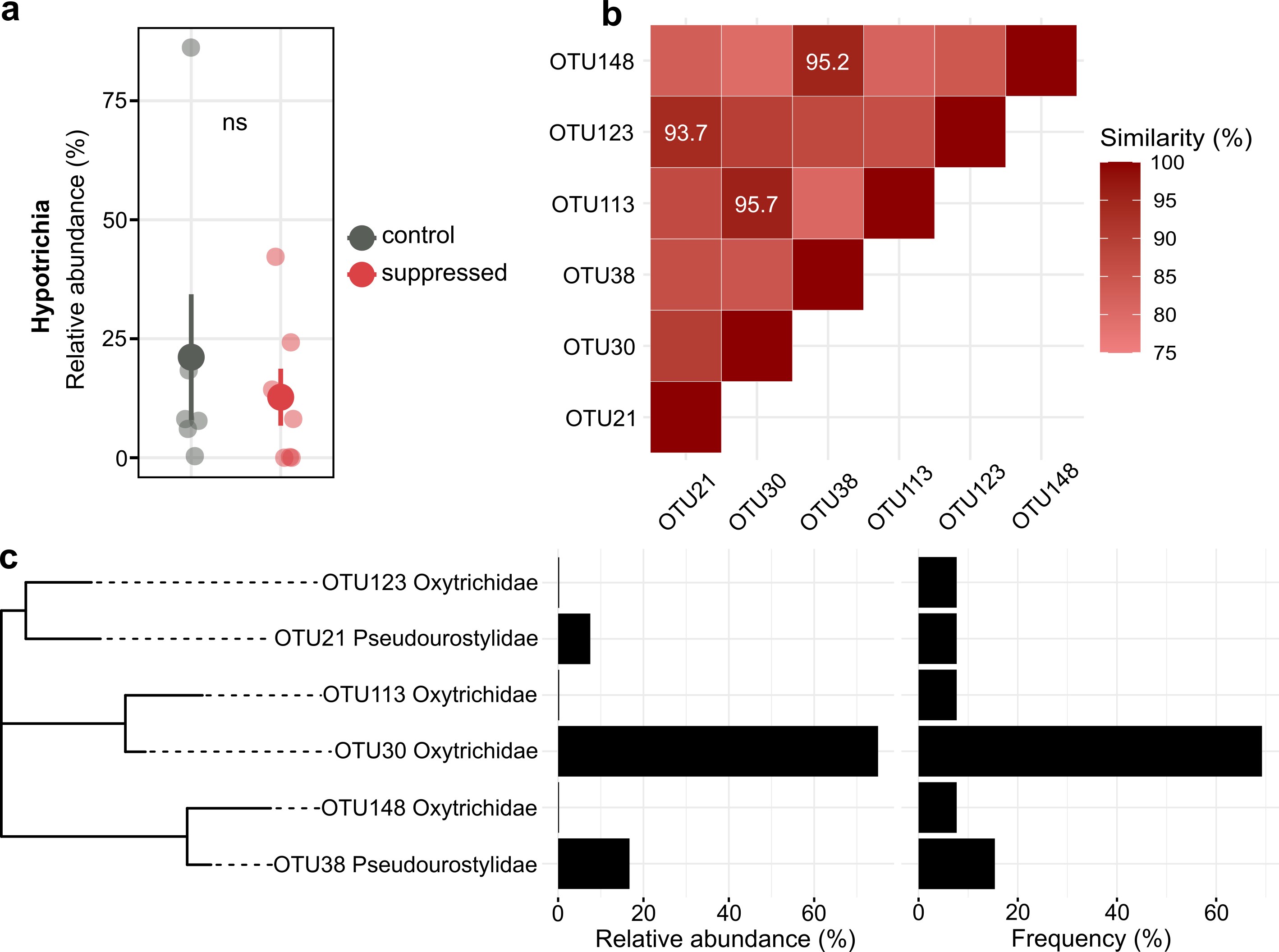

Supplement: Figure_S7_wraf253 [file figure_s7_wraf253.jpeg]

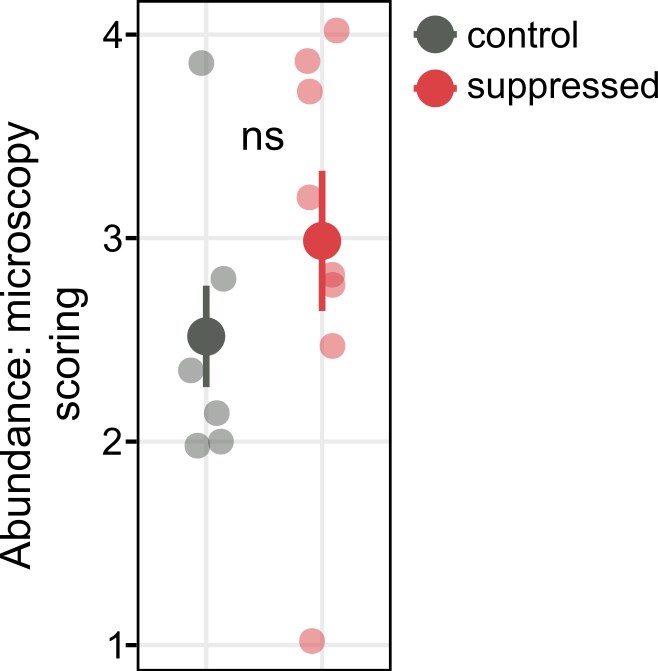

Supplement: Figure_S8_wraf253 [file figure_s8_wraf253.jpeg]

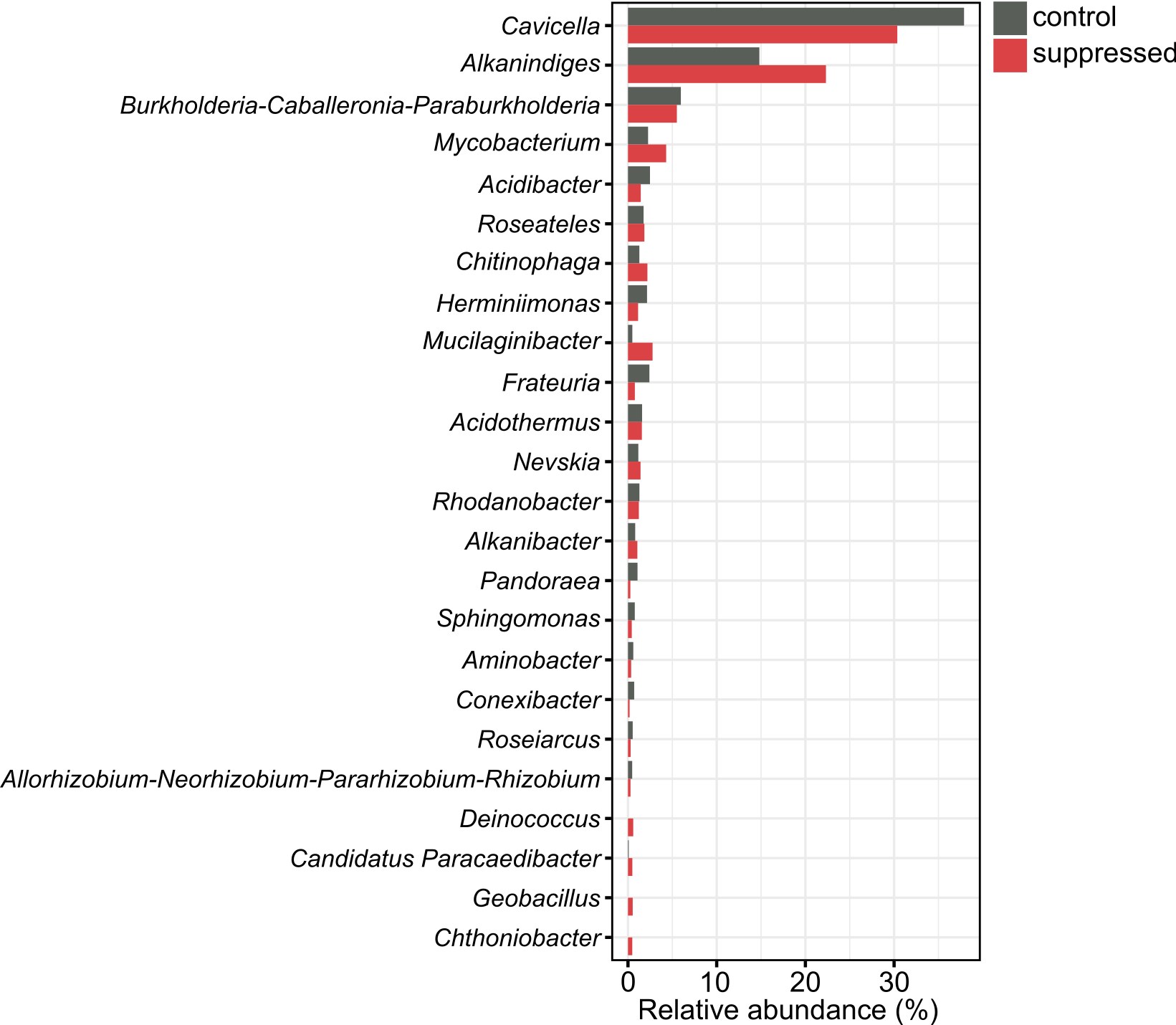

Supplement: Figure_S9_wraf253 [file figure_s9_wraf253.jpeg]

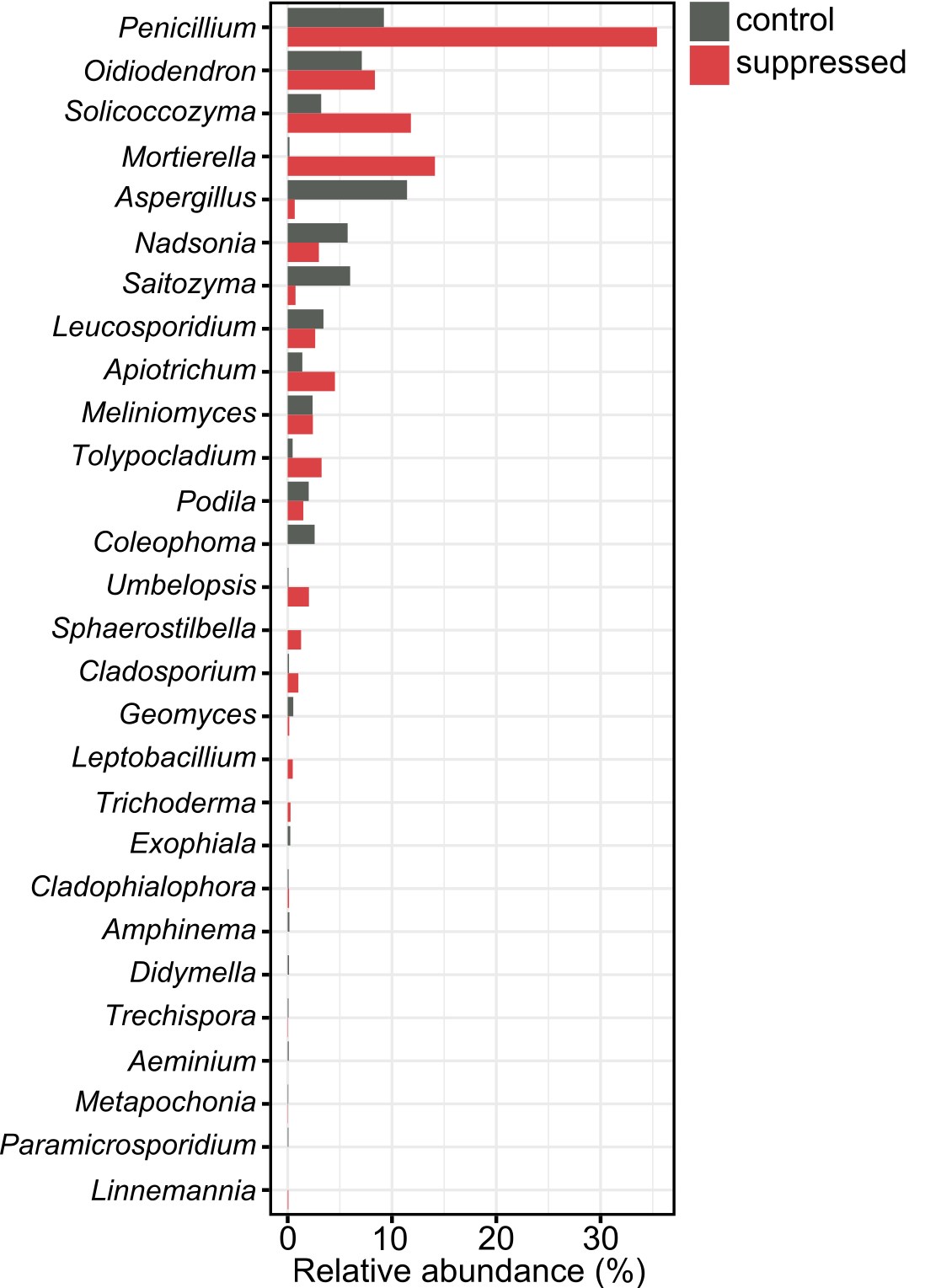

Supplement: Figure_S10_wraf253 [file figure_s10_wraf253.jpeg]

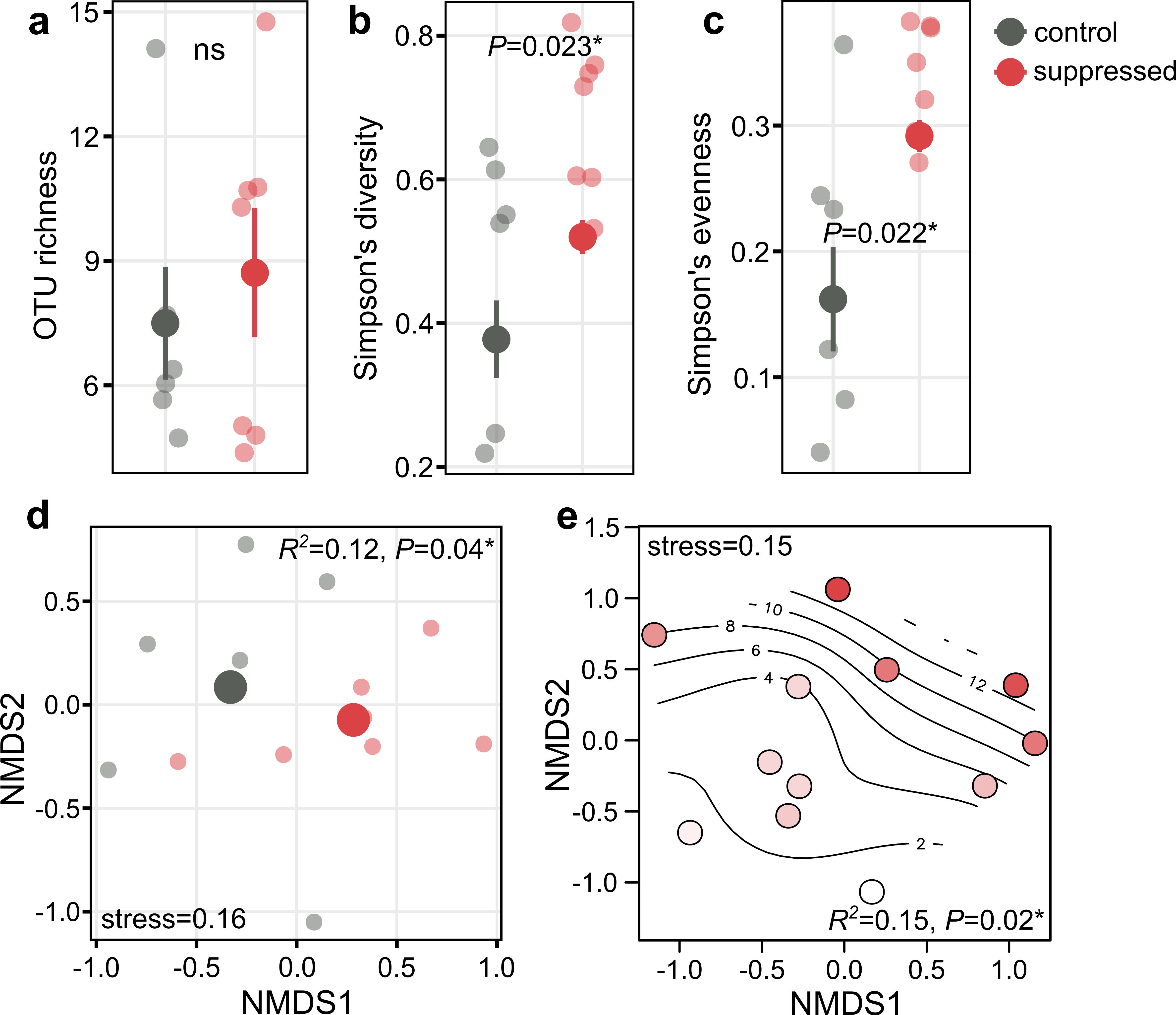

Supplement: Figure_S11_wraf253 [file figure_s11_wraf253.jpeg]

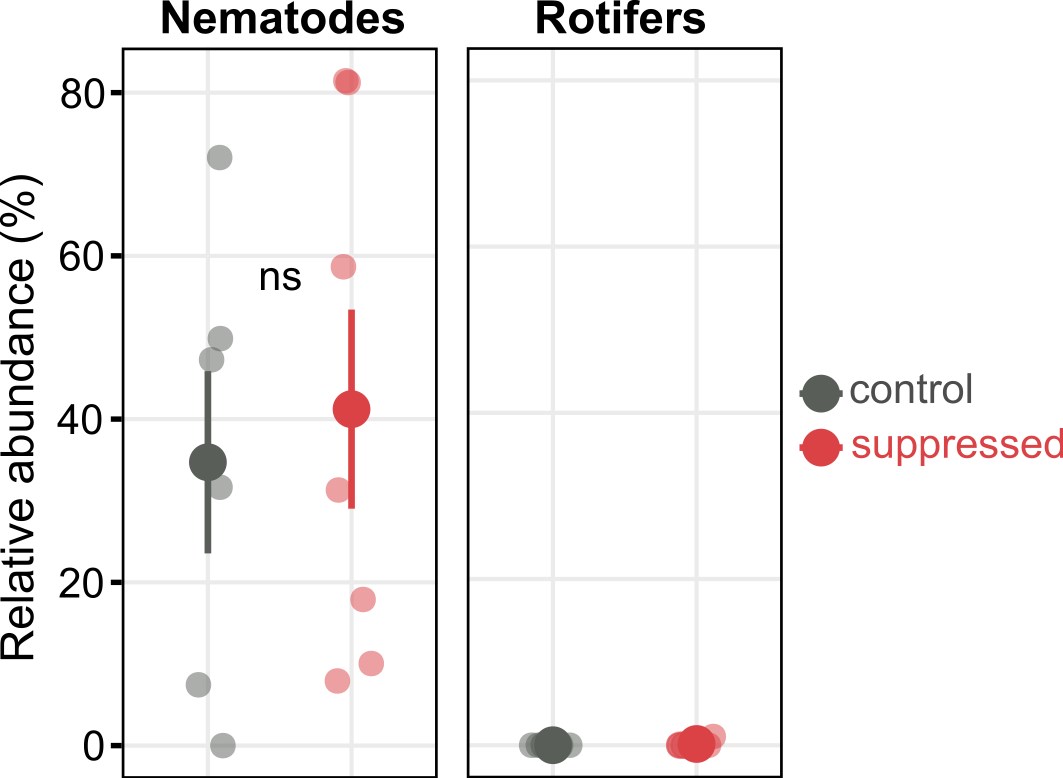

Supplement: Figure_S12_wraf253 [file figure_s12_wraf253.jpeg]

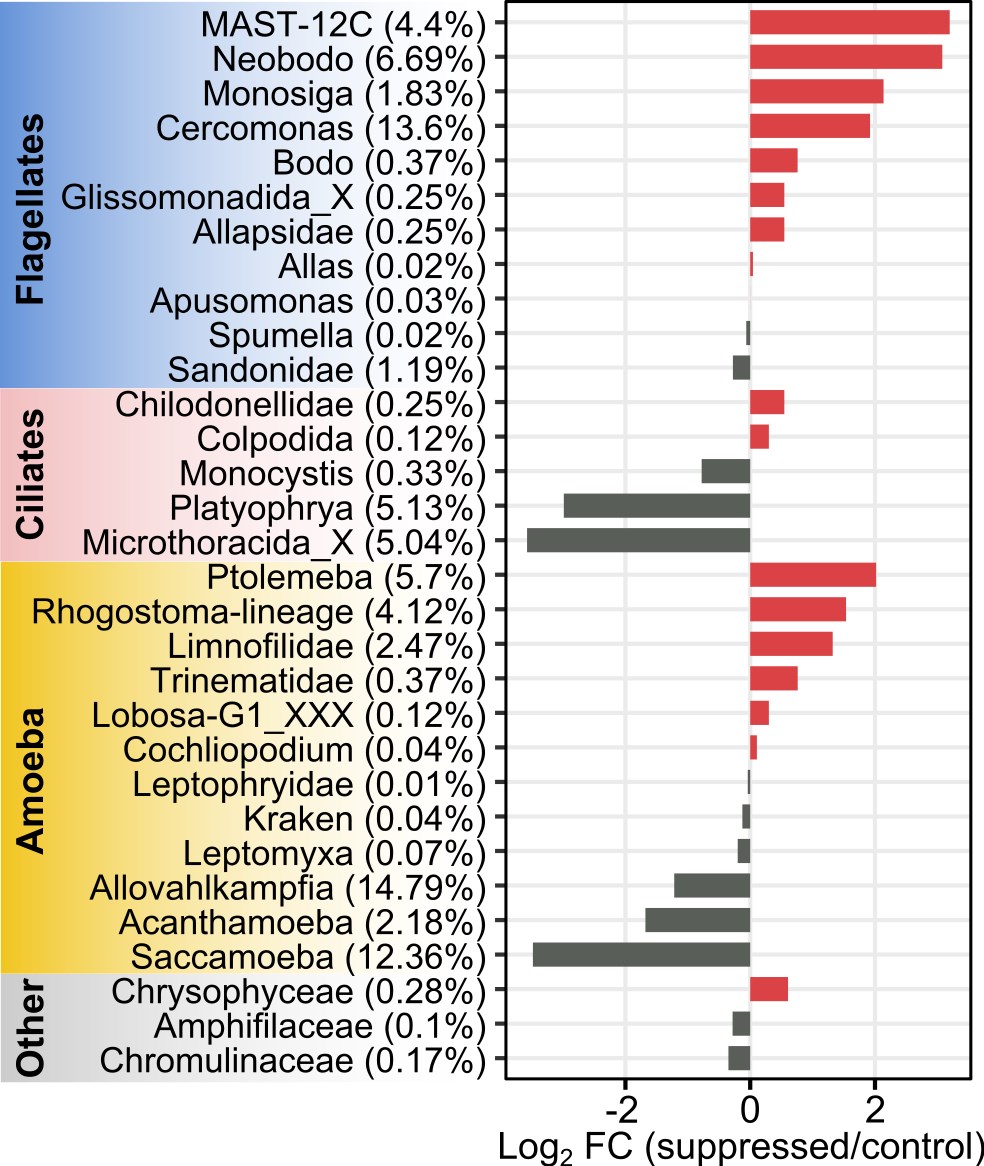

Supplement: Figure_S13_wraf253 [file figure_s13_wraf253.jpeg]

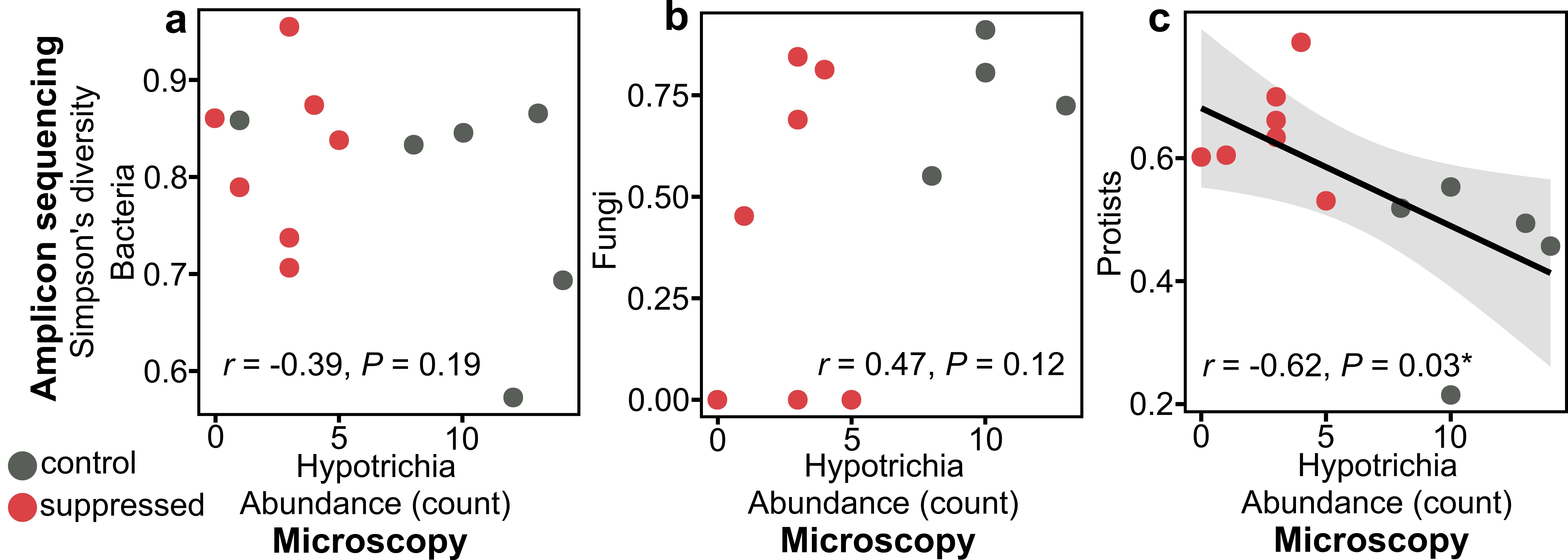

Supplement: Figure_S14_wraf253 [file figure_s14_wraf253.jpeg]
