# Supplementary material for: Keystone protist suppression triggers mesopredator release and biotic homogenization in complex soil microbial communities
Source: ISME J. 2025 Nov 14;19(1):wraf253. doi: 10.1093/ismejo/wraf253 (PMC12676721; doi:10.1093/ismejo/wraf253)
Supplement: Supplementary_material_Legends_wraf253 [file supplementary_material_legends_wraf253.docx]

**Supplementary material**

**Figure captions**

**Table S1.** Summary of chips used across experiments, detailing treatments and reasons for exclusion.

**Table S2.** Taxonomic assignments for protist morphogroups.

**Figure S1.** (a) Design schematic of the chips used in the study, comprising a 14,720 µm × 5000 µm cuboid structure with a pillar network (175 µm center-to-center spacing); (b) Representative microscopy image of a chip bonded to a glass coverslip, inoculated with fungal necromass and soil.

**Figure S2.** Effects of microscale UV suppression on 97 protist individuals in soil chips, showing post‑treatment life status (alive vs. dead) as a function of cell size and morphogroup.

**Figure S3.** Representative microscopy images showing Hypotrichia in chips inoculated with forest topsoil at 72, 99, and 112 s following a 5 s exposure to 395 nm excitation light (CoolLED pE300-White MB illumination). Yellow arrows indicate post-senescence vacuole formation. Scale bar = 50 µm.

**Figure S4.** Representative microscopy images illustrating bacterial abundance scoring in chips, ranging from 1 (very low abundance) to 5 (very high abundance). Scale bar = 40 µm.

**Figure S5.** Microscopy image demonstrating bacterial counting by deep-learning algorithm (Zou et al., 2024 [24]). Light blue squares indicate counted bacterial cells; the dark red square indicates a bacterial cluster excluded from the analysis. Scale bar = 40 µm.

**Figure S6.** Representative microscopy images for counting protist morphogroups: (a) Hypotrichia (suppressed), (b) non-Hypotrichia ciliates, (c) amoebae, (d) flagellates, and (e) fungal hyphae. Scale bar = 20 µm.

**Figure S7.** Hypotrichia abundance and identification: (a) Relative abundance based on 18S rRNA gene amplicon sequencing (dark dots are means ± SE, lighter dots represent individual chips, statistical comparisons via t-test); (b) 18S rRNA gene sequence similarity matrix among six Hypotrichia-assigned OTUs; (c) Neighbor-Joining phylogenetic tree showing sequence similarity, relative abundances (relative to total Hypotrichia), and frequencies across chips.

**Figure S8.** Effects of Hypotrichia suppression on bacterial abundance from microscopy scoring (1–5 scale, see Figure S2). Dark dots represent means ± SE; lighter dots individual chips. Statistical comparisons via t-tests or Welch’s tests.

**Figure S9.** Relative abundance of dominant bacterial genera by treatment (control vs. Hypotrichia suppression).

**Figure S10.** Relative abundance of dominant fungal genera by treatment (control vs. Hypotrichia suppression).

**Figure S11.** Effects of Hypotrichia suppression on protist communities assessed via 18S rRNA gene amplicon sequencing with Hypotrichia OTU retained. (a) OTU richness, (b) Simpson’s diversity, and (c) Simpson’s evenness (large dots means ± SE; small dots individual chips; statistical comparisons by t-tests or Welch’s tests). (d) Protist community composition (NMDS with Bray–Curtis dissimilarity; large dots centroids, small dots individual communities). (e) NMDS with Hypotrichia counts at day 20 as a continuous variable (circles colored by Hypotrichia abundance, darker indicates higher abundance; segments indicate specific counts per chip). Treatment effects tested via PERMANOVA (**P* < 0.05, ***P* < 0.01, ****P* < 0.001).

**Figure S12.** Relative abundance of nematodes and rotifers (compared to total protist OTUs) by treatment (control vs. Hypotrichia suppression); dark dots show means ± SE, lighter dots individual chips. Nematode abundances compared by t-test; rotifer abundance not statistically analyzed due to single-sample presence.

**Figure S13.** Differences in relative abundances of protist OTUs between control and suppressed treatments. OTUs summed at genus or higher taxonomic level when genus-level data was unavailable; numbers in brackets indicate overall relative abundance in 18S rRNA gene amplicon sequencing dataset.

**Figure S14.** Pearson’s correlations between Simpson’s diversity indices of (a) bacterial, (b) fungal, and (c) protist communities and Hypotrichia abundance (day 20, microscopy-based). Asterisks denote significance (**P* < 0.05, ***P* < 0.01, ****P* < 0.001).

**Video S1.** Hypotrichia foraging behavior within experimental chips, highlighting inoculated fungal necromass fragments (light brown/yellowish).

**Video S2.** Hypotrichia suppression demonstrated after 5-second exposure to 395 nm excitation light at 100% intensity.

**Dataset 1.** Hypotrichia counts per chip over time (days post-inoculation), based on microscopy.

**Dataset 2.** Bacterial, fungal, and protist morphogroups per chip at 20 days post-inoculation, based on microscopy.

**Dataset 3.** Bacterial abundance at 20 days post-inoculation, based on a deep learning algorithm.

**Dataset 4.** Bacterial abundance at 20 days post-inoculation, based on 16S rRNA gene qPCR.
