# Supplementary material for: Keystone protist suppression triggers mesopredator release and biotic homogenization in complex soil microbial communities
Source: ISME J. 2025 Nov 14;19(1):wraf253. doi: 10.1093/ismejo/wraf253 (PMC12676721; doi:10.1093/ismejo/wraf253)
Supplement: Supplementary_Methods_wraf253 [file supplementary_methods_wraf253.docx]

**Supplementary Methods**

*Chip design and fabrication*

The microfluidic chip design consisted of a cuboid space measuring 14,720 μm in length, 500 μm in breadth, and 12 μm in height, within which circular pillars approximately 100 μm in diameter were arranged in a regular array with a center-to-center spacing of 175 μm (see Figure S1a). This relatively small chip format was chosen to allow rapid inspection at low magnification (40×) and to facilitate the identification and suppression of relatively large protists. Chips were fabricated by molding PDMS (Sylgard 184, Dow Corning) on a photoresist master. To produce the master, a negative photoresist Mr-DWL 5 (Micro Resist Technology GmbH) was spin-coated onto a glass plate at 1500 rpm for 60 s. This layer was soft baked on a hot plate by ramping the temperature from 50 °C to 90 °C, holding at 90 °C for 10 minutes. It was then patterned with a maskless aligner (Heidelberg Instruments), followed by a post-exposure bake at 90 °C for 10 minutes. The photoresist was then developed for 4 min in mr-Dev 600 (MicroChem) and rinsed with isopropanol (VWR International). A SAM (self assembled monolayer) of FDTS(Perfluorodecyltrichlorosilane) was deposited using a Fiji ALD from Veeco for anti sticking purpose. PDMS slabs were produced by mixing the PDMS base and curing agent (both Sylgard 184, Dow Corning, USA) in a 10:1 mass/mass ratio. The mixture was poured onto the master in a 4-mm-thick layer and degassed in a vacuum chamber at −25 kPa for 45 min. The PDMS was cured in an oven at 60 °C for 2.5 h. Once cooled, the PDMS was cut slightly larger than the patterned area, and an entrance was created by excising one long side of the PDMS slab. Finally, the PDMS slabs were bonded to cover slip. The cover slips (55 × 75 mm; Thermo Scientific) were cleaned sequentially with acetone and isopropanol. Both the PDMS and coverslips were treated separately in an oxygen plasma chamber (Diener Electronic Zepto). The coverslip alone was exposed under UV-assisted plasma for 1 min, after which the PDMS and coverslip were plasma-treated together for 22 s. Immediately after treatment, the activated surfaces were brought together and gently pressed in the central region of the chip.

*Microscale UV-suppression non-target effect assay*

To test for potential non‑target effects of the UV suppression protocol, we prepared a test chip inoculated with the same soil and fungal necromass used in the main experiment and randomly applied the identical exposure used for Hypotrichia suppression: UV‑range excitation light (380–405 nm; CoolLED pE300‑White MB) at 100% intensity for 5 s at 400×. After exposure, we observed treated protist cells and recorded membrane rupture; cells showing no motility and/or no intracellular organelle movement within 30 s post‑exposure were scored as dead. Among the 97 protist cells treated, none exhibited membrane rupture (as seen in Hypotrichia), and only two cells showed apparent death; both were among the largest cells, which are rare in our chips and were avoided in the same field as Hypotrichia during the main suppression procedure (Figure S2).

*eDNA extraction*

After video recording on day 20, any soil on each chip was carefully removed. The chip contact area with soil was cleaned multiple times using a cotton swab moistened with molecular-grade water followed by a dry cotton swab to remove residual soil or necromass particles, thereby minimizing soil eDNA contamination. Using tweezers, the chip was detached from the coverslip, flipped over, and transferred to a Petri dish. The glass coverslip area that had been in contact with the chip was swabbed with a moistened cotton swab, and the tip was immediately placed into an extraction tube of the PowerSoil Pro Kit (Qiagen, Hilden, Germany). Using a sterile scalpel blade, the edges of the chip were excised to remove potential surface contaminants, and the remaining portion—representing the imprint of the pillar array in the PDMS—was transferred directly into the extraction tube. This procedure ensured that microbial cells adhering to both the coverslip and PDMS were collected. Genomic DNA was then extracted according to the PowerSoil Pro Kit manufacturer’s protocol, with a final elution volume of 50 μL. Three non-inoculated chips were processed identically as negative controls.

*Bacterial abundance quantification using a deep‑learning algorithm*

We used Ultralytics’ YOLOv11l deep‑learning segmentation model (Jocher et al., 2023) to automate bacterial counting. Following the methodology of Zou et al. [24], we expanded and curated the training set by pooling images from multiple Soil Chip experiments to improve accuracy and generalizability. Model training was conducted on supercomputers provided by the National Academic Infrastructure for Supercomputing in Sweden (NAISS) at the Chalmers Centre for Computational Science and Engineering (C3SE), utilizing four NVIDIA A100 GPUs. High‑resolution images (2,424 × 2,424 pixels) acquired with a Nikon Ti2‑E inverted light microscope were processed using a SAHI‑inspired Python library (Kolesnikov, 2025) to enable detection of small objects while balance field of view and computational load. The library tiles each large image into overlapping patches, performs detection on each patch independently, and then merges the results back into the original image coordinate space. While the algorithm is capable of identifying bacterial clusters and dividing cells, it sometimes misclassifies small necromass fragments (used here as the carbon and nutrient source) as bacterial cells. To avoid this confounding effect, we limited the analysis to individual bacterial cells. We further applied an inclusion cutoff on maximum cell length of 0.3–5 µm [24].

*Microbial qPCR*

Bacterial abundance was quantified via qPCR on a Stratagene Mx3005P PCR machine (Agilent Technologies), targeting the bacterial 16S rRNA gene with the 968F/1401R primer set (Cébron et al., 2008). Each 20 μL qPCR reaction contained 4 μL of template DNA, a standard 16S bacterial double-stranded DNA template (ranging from 10⁹ to 10³ gene copies μL⁻¹) or molecular-grade water as a negative control, and SsoAdvanced Universal Inhibitor-Tolerant Supermix (Bio-Rad, Hercules, CA, USA). The amplification protocol included an initial denaturation at 95 °C for 5 min, followed by 40 cycles of 20 s at 95 °C, 30 s at 56 °C, and 60 s at 72 °C. Primer specificity was confirmed via melting curve analysis from 70 °C to 95 °C, increasing at 0.3 °C s⁻¹. All samples were run in technical duplicates, and results were averaged to obtain the bacterial 16S rRNA gene copy number per chip. An attempt to quantify fungal abundance using qPCR with the FR1/FF390 primer set (Chemidlin Prévost-Bouré et al., 2011) was unsuccessful, as all samples fell outside the standard curve very likely due to low fungal genomic DNA concentrations.

*High-throughput amplicon sequencing*

To characterize microbial community structure within the chips, high-throughput sequencing (HTS) of taxonomic markers for bacteria, fungi, and protists was performed. Sixteen chip samples (from the 20 initially inoculated, with four excluded as previously noted) and three negative control chips were analyzed. Bacterial community structure was assessed by amplifying the V4 region of the 16S rRNA gene using the 515F–806R primers (Caporaso et al., 2012). Fungal community structure was characterized by targeting the ITS2 region with the 5.8S-Fun and ITS4-Fun primers (Taylor et al., 2017). For evaluating protist community structure, the 18S rRNA gene was amplified using the 616*f–1132r primer pair (Hugerth et al., 2014), which captures a broad taxonomic range of eukaryotes (Vaulot et al., 2022). First-round PCRs (20 μL reactions) contained 10 μL of Phusion Hot Start II High-Fidelity PCR Master Mix (Thermo Scientific, Waltham, MA, USA), 0.5 μL of each 20 mM primer, and 10 μL of template DNA. Preliminary tests indicated that replacing the water in the PCR mix entirely with template DNA improved amplification from the limited number of microbial cells in the chips. The thermocycling conditions were as follows: an initial denaturation at 98 °C for 30 s; then 39 cycles of 98 °C for 30 s, annealing at 50 °C (16S rRNA gene), 55 °C (ITS region), or 52 °C (18S rRNA gene) for 30 s, and extension at 72 °C for 30 s; with a final extension at 72 °C for 10 min. A second PCR was performed to add unique Golay barcodes and sequencing adapters. PCR products were purified and normalized using the Charm Just-a-Plate Purification and Normalization Kit (Charm Biotech, San Diego, CA, USA) and pooled at equimolar concentrations. Sequencing was conducted on an Illumina MiSeq platform using 2 × 300 bp chemistry at the University of Minnesota Genomics Center.

Sequencing data for 16S rRNA gene, ITS region, and 18S rRNA gene amplicons were processed using the DADA2 pipeline in R (Callahan et al., 2016). For the 18S rRNA gene and ITS region datasets, only forward reads (R1) were used to mitigate data loss due to poor R2 quality in combination with longer amplicon that requires relative high-quality score for efficient paired-end merging; paired-end merging was used for the 16S dataset. Raw fastq files were filtered to remove reads with ambiguous bases (maxN = 0), and primers were removed using Cutadapt (Martin, 2011). Quality filtering was applied with the following parameters: for 18S rRNA gene, a truncation length of 210 bp and maxEE = 25; for ITS region, truncLen = 210 bp and maxEE = 8; and for 16S rRNA gene, forward and reverse reads were truncated at 230 and 150 bp with maxEE values of 8 and 12, respectively. Error rates were learned, and amplicon sequence variants (ASVs) were inferred using DADA2, with chimeric sequences removed using removeBimeraDenovo. Taxonomy was assigned using the assignTaxonomy function with the PR2 database for 18S rRNA gene (Vaulot et al., 2022), the UNITE database for ITS region (Abarenkov et al., 2024), and the SILVA database for 16S rRNA gene (Quast et al., 2012). ASV sequences were then aligned using DECIPHER’s AlignSeqs, a distance matrix computed, and sequences clustered into operational taxonomic units (OTUs) at a 97% similarity threshold using DECIPHER’s TreeLine.

Following OTU clustering, OTU tables were run through additional quality control. OTUs detected in the three non-inoculated control chips were removed from the chip samples (11 OTUs for bacteria, 3 for fungi, and 3 for protists). Additionally, OTUs lacking kingdom-level taxonomic information were blasted against NCBI to confirm affiliation with the expected kingdom (bacteria, fungi, or protists), and those failing to match were omitted (8 OTUs for bacteria, 11 for fungi, and 3 for protists). OTUs corresponding to organelles (e.g., chloroplasts, mitochondria) were also removed (9 OTUs from the bacterial dataset). For the protist dataset, further filtering removed 72 bacterial OTUs, 35 fungal OTUs, 37 metazoan OTUs, and 1 plant (*Picea*) OTU. Metazoan OTUs were subsequently subset between nematodes and other microscopic animals (in this case, rotifers) to assess their potential impact on the results. The bacterial and fungal OTU tables were normalized by rarefaction using “rrarefy function” in R Vegan package (Oksanen et al., 2015) to 1716 and 2578 reads, respectively; two chips were removed from the bacterial analysis due to low sequence counts (533 and 624 reads) and three chips were excluded from the fungal analysis (with 0, 1, and 5 reads). To examine the effects of Hypotrichia suppression on protist community composition and diversity, all Hypotrichia sequences were removed from the protist dataset before the analyses. Due to the overall low read depth (average ~1060 sequences), normalization of this dataset was performed using relative abundances rather than rarefaction. One chip was further omitted because it yielded only a single 18S OTU, despite microscopy indicating a more diverse protist community. A summary of the chips retained for the community analyses is provided in Table S1. Finally, to facilitate direct comparison between microscopy and metabarcoding results, protist OTUs were regrouped into functional groups based on taxonomic information (see Table S2).

Five chips were excluded from microscopy analyses due to technical issues: one control chip (chip 18) exhibited a coverslip fissure during manipulation on day 20 and was used only for eDNA-based techniques; one suppression chip (chip 19) was omitted after PDMS unbonding was observed in the top left corner early during the experiment; and three additional chips (chips 4 and 5 from the suppression group, and chip 13 from the control group) were excluded due to very low Hypotrichia colonization (see Table S1 for a summary of the chips retained for analysis).

**References**

Abarenkov K, Nilsson RH, Larsson KH, et al. The UNITE database for molecular identification and taxonomic communication of fungi and other eukaryotes: sequences, taxa and classifications reconsidered. Nucleic Acids Res 2024;52:D791–D797. <https://doi.org/10.1093/nar/gkad1039>

Aleklett K, Ohlsson P, Bengtsson M, et al. Fungal foraging behaviour and hyphal space exploration in micro-structured Soil Chips. ISME J 2021;15:1782–1793. <https://doi.org/10.1038/s41396-020-00886-7>

Caporaso JG, Lauber CL, Walters WA, et al. Ultra-high-throughput microbial community analysis on the Illumina HiSeq and MiSeq platforms. ISME J 2012;6:1621–1624. <https://doi.org/10.1038/ismej.2012.8>

Callahan BJ, McMurdie PJ, Rosen MJ, et al. DADA2: High-resolution sample inference from Illumina amplicon data. Nat Methods 2016;13:581–583. <https://doi.org/10.1038/nmeth.3869>

Cébron A, Norini MP, Beguiristain T, Leyval C. Real-Time PCR quantification of PAH-ring hydroxylating dioxygenase (PAH-RHDα) genes from Gram positive and Gram negative bacteria in soil and sediment samples. J Microbiol Methods 2008;73:148–159. <https://doi.org/10.1016/j.mimet.2008.01.009>

Chemidlin Prévost-Bouré N, Christen R, Dequiedt S, et al. Validation and application of a PCR primer set to quantify fungal communities in the soil environment by real-time quantitative PCR. PLoS ONE 2011;6:e24166. <https://doi.org/10.1371/journal.pone.0024166>

Hugerth LW, Muller EEL, Hu YOO, et al. Systematic design of 18S rRNA gene primers for determining Eukaryotic diversity in microbial consortia. PLoS ONE 2014;9:e95567. <https://doi.org/10.1371/journal.pone.0095567>

Jocher, G., Qiu, J., & Chaurasia, A. (2023). Ultralytics YOLO (Version 8.0.0) [Computer software]. https://github.com/ultralytics/ultralytics

Kolesnikov, D. (2025) ‘Koldim2001/YOLO-Patch-Based-Inference’. Available at: https://github.com/Koldim2001/YOLO-Patch-Based-Inference (Accessed: 19 June 2025).

Mafla-Endara PM, Arellano-Caicedo C, Aleklett K, et al. Microfluidic chips provide visual access to in situ soil ecology. Commun Biol 2021;4:889. <https://doi.org/10.1038/s42003-021-02379-5>

Martin, M. (2011). Cutadapt removes adapter sequences from high-throughput sequencing reads. EMBnet. journal 17, 10–12.

Oksanen, J., Blanchet, F. G., Kindt, R., Legendre, P., Minchin, P. R., O’hara, R. B., ... & Oksanen, M. J. 2013. Package ‘vegan’. Community ecology package, version, 2, 1–295.

Quast C, Pruesse E, Yilmaz P, et al. The SILVA ribosomal RNA gene database project: improved data processing and web-based tools. *Nucleic Acids Res* 2012;**41**:D590–D596. <https://doi.org/10.1093/nar/gks1219>

Taylor DL, Walters WA, Lennon NJ, et al. Accurate estimation of fungal diversity and abundance through improved lineage-specific primers optimized for Illumina amplicon sequencing. Appl Environ Microbiol 2016;82:7217–7226. <https://doi.org/10.1128/AEM.02576-16>

Vaulot D, Geisen S, Mahé F, et al. pr2‐primers: An 18S rRNA primer database for protists. Mol Ecol Resour 2022;22:168–179. <https://doi.org/10.1111/1755-0998.13465>

Zou H, Sopasakis A, Maillard F, et al. Bacterial community characterization by deep learning aided image analysis in soil chips. Ecol Inform 2024;81:102562. <https://doi.org/10.1016/j.ecoinf.2024.102562>
